# A world of taxonomic pain: cryptic species, inexplicable host-specificity, and host-induced morphological variation among species of *Bivesicula* Yamaguti, 1934 (Trematoda: Bivesiculidae) from Indo-Pacific Holocentridae, Muraenidae and Serranidae

**DOI:** 10.1017/S0031182022000282

**Published:** 2022-05

**Authors:** Thomas H. Cribb, Rodney A. Bray, Jean-Lou Justine, James Reimer, Pierre Sasal, Sho Shirakashi, Scott C. Cutmore

**Affiliations:** 1 School of Biological Sciences, The University of Queensland, St Lucia, Queensland 4072, Australia; 2 Department of Life Sciences, Natural History Museum, Cromwell Road, London SW7 5BD, UK; 3 ISYEB, Institut de Systématique Évolution Biodiversité, UMR7205 MNHN, CNRS, EPHE, UPMC, Université des Antilles, Muséum National d'Histoire Naturelle, 43 Rue Cuvier, 75005 Paris, France; 4 Molecular Invertebrate Systematics and Ecology, Faculty of Science, University of the Ryukyus, 1 Senbaru, Nishihara, Okinawa 903-0213, Japan; 5 CRIOBE, USR3278-EPHE/CNRS/UPVD/PSL, University of Perpignan Via Domitia, 52 Avenue Paul Alduy, 66860 Perpignan, France; 6 Aquaculture Research Institute, Kindai University, Shirahama 3153, Wakayama 649-2211, Japan

**Keywords:** Cryptic species, distribution, host-induced variation, host-specificity, phylogeny, taxonomy, Trematoda

## Abstract

The taxonomy of species of *Bivesicula* Yamaguti, 1934 is analysed for samples from holocentrid, muraenid and serranid fishes from Japan, Ningaloo Reef (Western Australia), the Great Barrier Reef (Queensland), New Caledonia and French Polynesia. Analysis of three genetic markers (*cox*1 mtDNA, ITS2 and 28S rDNA) identifies three strongly supported clades of species and suggests that *Bivesicula* as presently recognized is not monophyletic. On the basis of combined morphological, molecular and biological data, 10 species are distinguished of which five are proposed as new. *Bivesicula* Clade 1 comprises seven species of which three are effectively morphologically cryptic relative to each other; all seven infect serranids and four also infect holocentrids. *Bivesicula* Clade 2 comprises three species of which two are effectively morphologically cryptic relative to each other; all three infect serranids and one also infects a muraenid. *Bivesicula* Clade 3 comprises two known species from apogonids and a pomacentrid, and forms a clade with species of *Paucivitellosus* Coil, Reid & Kuntz, 1965 to the exclusion of other *Bivesicula* species. Taxonomy in this genus is made challenging by the combination of low resolving power of ribosomal markers, the existence of regional *cox*1 mtDNA populations, exceptional and unpredictable host-specificity and geographical distribution, and significant host-induced morphological variation.

## Graphical abstract

**Figure:**
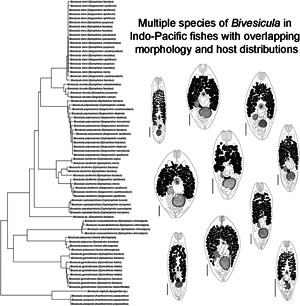

## Introduction

A key component of the understanding of biodiversity of any group is geographical distribution. In their review of the biodiversity of trematodes of fishes of the Indo-west Pacific (IWP), Cribb *et al*. (2016) found that ‘understanding of the geographical distribution of trematode species in the IWP is especially wanting’. They pointed out that records for the region are heavily concentrated in just a few regions (especially off Hawaii, India, Japan and the Great Barrier Reef) and that 87% of known species have been reported no more than five times. The lack of records over range is exacerbated by the lack of molecular testing of parasite identity, in a group seemingly especially prone to cryptic speciation (Poulin, 2011; Pérez-Ponce de León and Poulin, 2018). Recently, a handful of studies has begun to redress this ignorance with combined morphological and molecular studies of specific taxa from widely separated IWP sites. These studies show that species from multiple families can be interpreted as geographically widespread, although often with distinct regional populations (McNamara *et al*., 2014; Bray *et al*., 2018; Bray *et al*., 2022; Cutmore *et al*., 2021; Huston *et al*., 2021). Less frequently, there has been evidence of separate closely related species in the same fish species across range (Cribb *et al*., 2014; Martin *et al*., 2018). These studies, especially the recognition of regional genetic variation, have crystallized problems on the basis of species recognition, leading Bray *et al*. (2022) to propose objective criteria for recognition of trematode species over range in the light of molecular data. This approach is applied here to trematodes of the family Bivesiculidae Yamaguti, 1934, a group for which molecular approaches have been little used (Trieu *et al*., 2015; Atopkin *et al*., 2017) and not at all over range. The Bivesiculidae is a small family of exclusively fish parasites occurring in a wide range of marine fish families (Cribb, 2002). This study aims to differentiate IWP species objectively and to characterize their host-specificity and geographical distribution.

## Materials and methods

### Sample collection and morphology

Fishes were collected by spear, seine net, line or purchased from fish markets at localities off Australia, Japan, French Polynesia and New Caledonia (Table 1). Digeneans were collected from freshly killed fish as described by Cribb and Bray (2010), fixed by being pipetted into nearly boiling saline, and immediately preserved in either formalin (early work) or 80% ethanol (more recent work). Some individual worms preserved in 80% ethanol were processed for both morphological and molecular analyses (hologenophores sensu Pleijel *et al*., 2008) and many specimens were also designated as paragenophores (specimens collected from the same host individual as sequenced specimens).

**Table 1.** Collection localities and geographical distribution of 10 species of *Bivesicula*

Locality	Co-ordinates	1	2	3	4	5	6	7	8	9	10
Gambier Archipelago, French Polynesia	23°7′S, 134°58′W								⬤		
Moorea, Society Archipelago, French Polynesia	17°30′S, 149°50′W								⬤		
Tubuai, Austral Archipelago, French Polynesia	22°23′S, 149°28′W								⬤		
New Caledonia	21°30′S, 165°30′E	⬤				⬤		⬤			
Heron Is., southern Great Barrier Reef	23°27′S, 151°55′E						⬤			⬤	
Lizard Is., northern Great Barrier Reef	14°40′S, 145°27′E	⬤	⬤		⬤					⬤	
Minabe fish market, Wakayama Prefecture, Japan	33°44′N, 135°19′E			⬤							
Tomari fish market, Naha, Okinawa Prefecture, Japan	26°13′N, 127°40′E		⬤	⬤				⬤			
Ningaloo Reef, northern Western Australia	22°42′S, 113°40′E		⬤								
Kedonganan Fish Market, Bali, Indonesia	8°45′S, 115°10′E										⬤

1. *B. cephalopholicola* n.sp.; 2.*B. claviformis*; 3.*B. gymnothoracis*; 4.*B. nana* n.sp.; 5.*B. novaecaledoniensis* n.sp.; 6.*B. obovata*; 7.*B. palauensis*; 8.*B. polynesiensis* n.sp.; 9.*B. sheni*; 10. *B.* ‘Bali'sp.

Whole mounts for morphological analysis were stained with Mayer's haematoxylin, dehydrated in a graded ethanol series, cleared in methyl salicylate and mounted in Canada balsam. Drawings were made using a drawing tube on an Olympus BX-53 (Tokyo, Japan) compound microscope. Morphometric data were taken with the same microscope with cellSens Standard imaging software. The long axis of the testis and the ovary rarely aligned with that of the body. To capture the full size of these organs the maximum detectable length and the maximum width perpendicular to that length were measured. The following abbreviations are used: MNHN, Museum National d'Histoires Naturelles, Paris, France; MPM, Meguro Parasitological Museum, Tokyo, Japan; NHMUK, Natural History Museum, London, UK; QM, Queensland Museum, Brisbane, Australia; WAM, Western Australian Museum, Perth, Australia. To comply with the recommendations set out in article 8.5 of the amended 2012 version of the International Code of Zoological Nomenclature (ICZN, 2012), details of the new species have been submitted to ZooBank and registered with Life Science Identifiers (LSID), which are provided in the taxonomic summaries.

### DNA sequencing and phylogenetic analysis

Total genomic DNA was extracted using phenol/chloroform extraction techniques (Sambrook and Russell, 2001). Three genetic markers were sequenced, the nuclear second internal transcribed spacer (ITS2 rDNA) and the large (28S) ribosomal subunit RNA coding regions and the mitochondrial *cox*1 region (*cox*1 mtDNA). The complete ITS2 rDNA region (with flanking 5.8S and 28S rDNA regions) was amplified using the primers 3S (5′-GGT ACC GGT GGA TCA CGT GGC TAG TG-3′; Morgan and Blair, 1995) and ITS2.2 (5′-CCT GGT TAG TTT CTT TTC CTC CGC-3′; Cribb *et al*., 1998), the partial D1-D3 28S rDNA region using LSU5 (5′-TAG GTC GAC CCG CTG AAY TTA AGC A-3′; Littlewood, 1994), and 1500R (5′-GCT ATC CTG AGG GAA ACT TCG-3′; Snyder and Tkach, 2001), and the partial *cox*1 mtDNA region using Dig_cox1Fa (5′-GCT ATC CTG AGG GAA ACT TCG-3′; Wee *et al*., 2017) and Dig_cox1R (5′-TCN GGR TGH CCR AAR AAY CAA AA-3′; Wee *et al*., 2017). In addition to the new *Bivesicula* specimens being studied, genetic data were also generated for three other species from archival samples, for comparative purposes: *B. neglecta* Trieu, Cutmore, Miller and Cribb, 2015 (*cox*1: OM456635; ITS2: OM523336; 28S: OM459986); *B. unexpecta* Cribb, Bray and Barker, 1994 (*cox*1: OM456679–80; ITS2: OM523355); and an undescribed species from Bali, *Bivesicula* sp. (*cox*1: OM456678; ITS2: OM523354).

PCR for the ITS2 rDNA and 28S rDNA regions was performed with a total volume of 20 *μ*L, consisting of 5 *μ*L of 5 × MyTaq reaction buffer (Bioline), 0.75 *μ*L of each primer, 0.25 *μ*L of Taq polymerase (Bioline MyTaq™ DNA polymerase), and 2 *μ*L of DNA template, made up to 20 *μ*L with Invitrogen™ ultraPURE™ distilled water. PCR for the *cox*1 mtDNA region was performed with a total volume of 20 *μ*L, consisting of 5 *μ*L of 5 × MyTaq reaction buffer, 2 *μ*L of each primer, 0.25 *μ*L of Taq polymerase and 4 *μ*L of DNA template, made up to 20 *μ*L with Invitrogen™ ultraPURE™ distilled water. Amplifications were carried out on a Takara TP-650 PCR thermocycler (Beijing, China). The following profile was used to amplify the ITS2 rDNA region: an initial single cycle of 95°C denaturation for 3 min, 45°C annealing for 2 min, 72°C extension for 90 s, followed by 4 cycles of 95°C denaturation for 45 s, 50°C annealing for 45 s, 72°C extension for 90 s, followed by 30 cycles of 95°C denaturation for 20 s, 52°C annealing for 20 s, 72°C extension for 90 s, with a final 72°C extension for 5 min. The following profile was used to amplify the 28S rDNA region: an initial 95°C denaturation for 4 min, followed by 30 cycles of 95°C denaturation for 1 min, 56°C annealing for 1 min, 72°C extension for 2 min, followed by a single cycle of 95°C denaturation for 1 min, 55°C annealing for 45 s, with a final 72°C extension for 4 min. The following profile was used to amplify the *cox*1 mtDNA region: an initial 94°C denaturation for 3 min, followed by 40 cycles of 94°C denaturation for 30 s, 50°C annealing for 30 s, 72°C extension for 30 s, with a final extension at 72°C for 10 min. Cycle sequencing of amplified DNA was carried out at the Australian Genome Research Facility with ABI Big Dye™ v.3.1 chemistry following the manufacturer's recommendations, using the same primers used for PCR amplification as well as the additional 28S primers 300F [5′-CAA GTA CCG TGA GGG AAA GTT G-3′; (Littlewood *et al*., 2000)] and ECD2 [5′-CCT TGG TCC GTG TTT CAA GAC GGG-3′; (Littlewood *et al*., 1997)]. Geneious^®^ version 10.2.6 (Kearse *et al*., 2012) was used to assemble and edit contiguous sequences.

ITS2 rDNA and *cox*1 mtDNA sequence data generated during this study were each aligned with MUSCLE in MEGA 11 (Tamura *et al*., 2021) using UPGMA clustering for iterations 1 and 2.ITS2 rDNA sequences generated during this study were aligned withrelevant sequences available on GenBank (KR092219–22, Trieu *et al*., 2015). The ends of each ITS2 rDNA fragment were trimmed for a final dataset of 453 base positions. The *cox*1 mtDNA alignment (474 base positions) was transferred to Mesquite v.3.31 (Maddison and Maddison, 2021), translated (Translation Table 9: Echinoderm/flatworm mitochondrial code) and inspected for internal stop codons. All codon positions in the *cox*1 mtDNA dataset were evaluated for substitution saturation, as well as non-stationarity caused by base composition bias. Substitution saturation was assessed using the ‘Test of substitution saturation by Xia *et al*.’ function (Xia *et al*., 2003; Xia and Lemey, 2009) as implemented in DAMBE v.7.2 (Xia, 2018); no significant substitution saturation was detected. Non-stationarity was assessed using the *χ*^2^ function in PAUP v.4.0 (Swofford, 2002); significant non-stationarity was not detected. Thus, all codons in the *cox*1 mtDNA dataset were used in downstream analyses. Pairwise differences were estimated for each dataset using the following conditions: ‘Variance Estimation Method = None’, ‘Model/Method = No. of differences’ and ‘Substitutions to Include = d: Transitions + Transversions’ and ‘Gaps/Missing Data Treatment = Pairwise deletion’. Unrooted Neighbour joining analyses were conducted using MEGA 11 for each dataset to explore species boundaries, with the following parameters: ‘Model/Method = No. of differences’, ‘Substitutions to Include = d: Transitions + Transversions’, ‘Gaps/Missing Data Treatment = Pairwise deletion’ and ‘Rates among Sites = Gamma Distributed’. Nodal support was estimated by performing 10 000 bootstrap replicates.

The partial 28S rDNA sequences generated during this study were aligned with sequences of related bivesiculids from GenBank (AY222181 and AY222183; Olson *et al*., 2003) (LN831716-17, Atopkin *et al*., 2017) using MUSCLE version 3.7 (Edgar, 2004) run on the CIPRES portal (Miller *et al*., 2010), with ClustalW sequence weighting and UPGMA clustering for iterations 1 and 2.The resultant alignment was refined using MESQUITE (Maddison and Maddison, 2021) and the ends of the alignment were trimmed for a final length of 1340 base positions. Bayesian inference and maximum likelihood analyses of the 28S rDNA dataset were conducted to explore relationships among these taxa. Bayesian inference analysis was performed using MrBayes version 3.2.7a (Ronquist *et al*., 2012) run on the CIPRES portal and maximum likelihood analysis using MEGA 11. The best nucleotide substitution model was estimated using jModelTest version 2.1.10. The Akaike Information Criterion (AIC) and Bayesian Information Criterion (BIC) predicted the TIM3 + Γ model as the best estimator; Bayesian inference and maximum likelihood analyses were conducted using the closest approximation to this model. Nodal support in the maximum likelihood analysis was estimated by performing 1000 bootstrap pseudoreplicates. Bayesian inference analysis was run over 10 000 000 generations (ngen = 10 000 000) with two runs each containing four simultaneous Markov chain Monte Carlo (MCMC) chains (nchains = 4) and every 1000th tree saved. Bayesian inference analysis used the following parameters: ‘nst = 6’, ‘rates = gamma’, ‘ngammacat = 4’, and the prior parameters of the combined dataset were set to ‘ratepr = variable’. Samples of substitution model parameters, and tree and branch lengths were summarized using the parameters ‘sump burnin = 3000’ and ‘sumt burnin = 3000’. Species of the Transversotrematidae were designated as outgroup taxa (KX186733, KX186736 and KX186743, Cutmore *et al*., 2016).

## Results

In total, 188 individuals of 15 species of Holocentridae and 807 individuals of 45 species of Serranidae from nine localities in the Indo-West Pacific were examined. In addition, bivesiculids from 275 individuals of 28 New Caledonian serranids collected by Justine *et al*. (2010) were examined. Infections of species of *Bivesicula* were found in 43 parasite/host/locality combinations. None of the species concerned were ever detected in any of thousands of individuals of other families of fishes, except for one species infecting a muraenid. Although some combinations of the specimens are morphologically distinctive, species boundaries and phylogenetic relationships were also explored with molecular data.

### *cox*1 mtDNA analysis

**Fig. 1.**
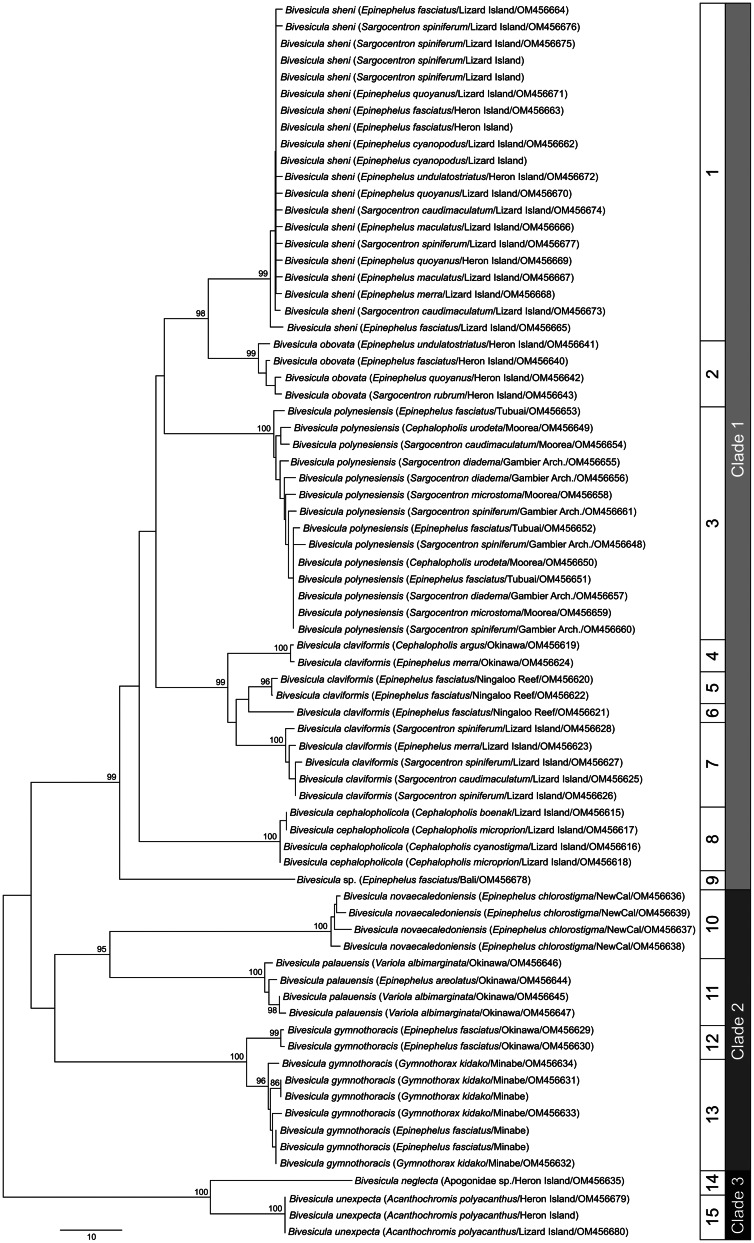
Phylogram from the unrooted Neighbour-joining analysis of the *cox*1 mtDNA dataset. Bootstrap support values are shown at the nodes, with values of <85 not shown. Numbers 1–15 indicate operational taxonomic units (OTUs). The scale bar indicates the number of base differences. NewCal, New Caledonia.

*cox*1 mtDNA data were generated, as far as possible, for at least two individuals of every available host/locality combination. A total of 74 *cox*1 mtDNA sequences were aligned without ambiguity or alignment gaps. An arbitrary criterion of 10 base position differences was set as potentially informative for the recognition of species or populations; these groups were treated initially as operational taxonomic units (OTUs). Differences at <10 base positions were interpreted as inconsequential intraspecific variation. On the basis of this criterion, 15 OTUs were detected (Fig. 1); the OTUs and final species level hypothesis are both labelled on the tree. Replication within these OTUs ranges from one (no replication) to 20. Three major clades (Clades 1, 2 and 3) are distinguished, two with high nodal support and one (Clade 2) with poor support; Clade 1 includes nine OTUs, Clade 2 includes four OTUs and Clade 3 includes two OTUs.

### ITS2 rDNA analysis

**Fig. 2.**
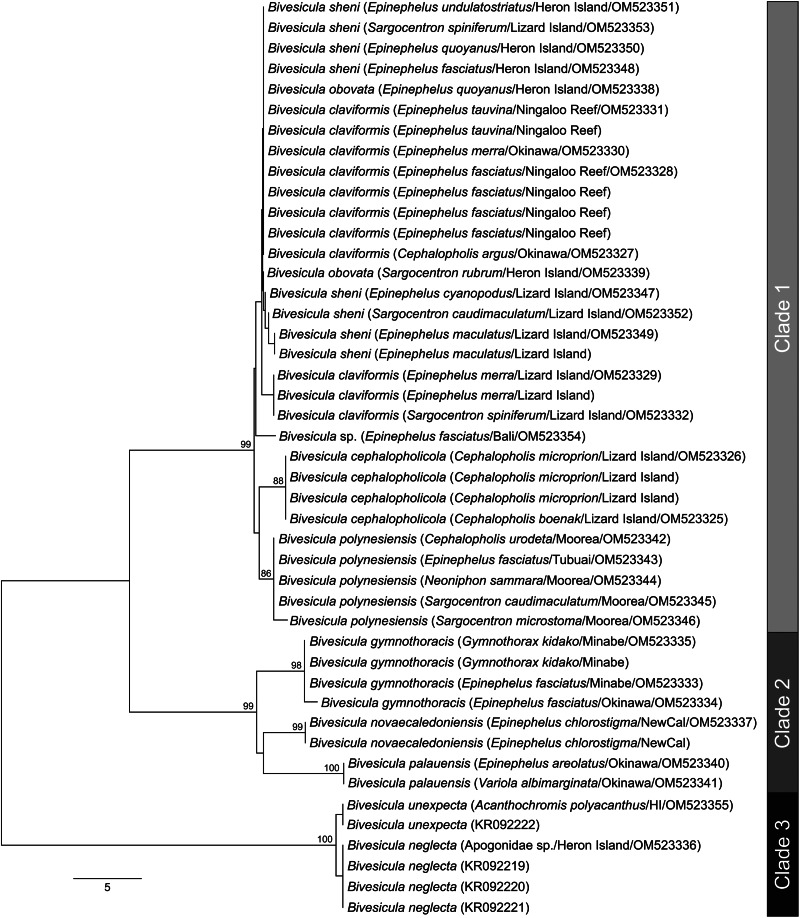
Phylogram from the unrooted Neighbour-joining analysis of the ITS2 rDNA dataset. Bootstrap support values are shown at the nodes, with values of <85 not shown. The scale bar indicates the number of base differences. HI, Heron Island; NewCal, New Caledonia.

ITS2 rDNA sequences were generated for, where possible, a minimum of two representatives from each *cox*1 OTU. The analysis of 41 sequences (Fig. 2) distinguished the same three major clades as the *cox*1 analyses, each with strong nodal support. Within Clade 1 (relating to nine *cox*1 OTUs), variation was exceptionally low, not exceeding four base positions. These distinctions are consistent with only four weakly differentiated OTUs. Within Clade 2, three well-supported clades were distinguished at 8–10 base positions. Within Clade 3, the two *cox*1 OTUs differed at a single base position.

### 28S rDNA analysis

**Fig. 3.**
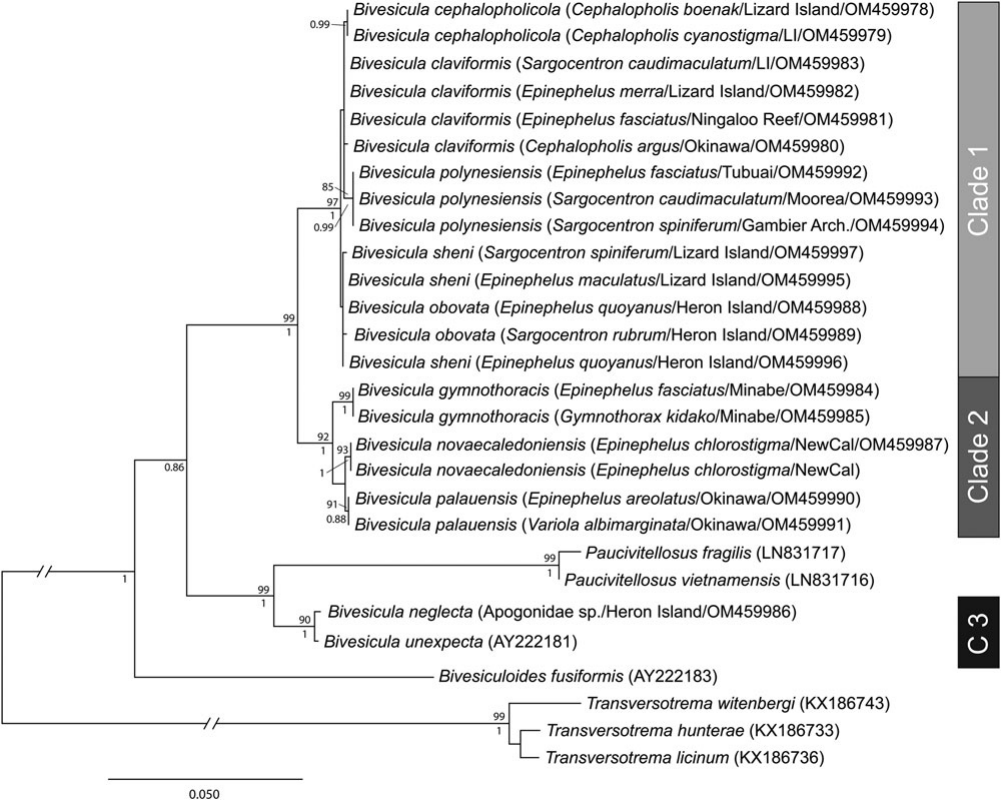
Relationships between species of *Bivesicula* and other members of the Bivesiculidae based on phylogenetic analysis of the 28S dataset. Maximum likelihood bootstrap support shown above the nodes and Bayesian inference posterior probabilities values are shown below; values of <85 and <0.85 not shown. The scale bar indicates expected number of substitutions per site. LI, Lizard Island; NewCal, New Caledonia.

28S rDNA sequences were generated for, where possible, the same two representatives of each *cox*1 OTU that were sequenced for ITS2 rDNA. Twenty-six sequences were analysed with other previously reported bivesiculid 28S rDNA sequence data. The results of this analysis (Fig. 3) again identified *Bivesicula* species as forming three highly supported clades. Sequences of species of Clades 1 and 2 differed at 27–29 base positions whereas those in Clade 3 differed from those in Clades 1 and 2 at 78–81 base positions. Clade 1 lineages showed only a low level of variation at a maximum of 4 or 5 base positions, which gave almost no useful resolution of OTUs. Clade 2 distinguished the same three OTUs as seen in the *cox*1 mtDNA and ITS2 rDNA analyses but with lower nodal support. Clade 3 formed a strongly supported clade with two species of *Paucivitellosus* to the exclusion of other species of *Bivesicula*. *Bivesiculoides fusiformis* Cribb, Bray and Barker, 1994 was identified as sister to all other sequenced bivesiculids.

### Morphology

A total of 331 slide specimens, including 30 hologenophores and 108 paragenophores were prepared and examined. These specimens corresponded to all of the OTUs referred to above except that there is no specimen relating to the single sequence from *Epinephelus fasciatus* (Forsskål) from off Bali. In addition to specimens corresponding to the molecular data set, multiple host–locality combinations for which no sequences were available were examined. These specimens were measured systematically, and drawings were made of at least one individual of each of the 15 OTUs identified by *cox*1 mtDNA sequencing.

Specimens relating to the three clades of species of *Bivesicula* identified by phylogenetic analyses are easily distinguished by the combination of the form of the pharynx which is large and robust in Clade 1 and proportionally far smaller in Clades 2 and 3.The two species of Clade 3 are distinguished from those of Clade 2 by their relatively small and rounded bodies with intestinal caeca exceeding the testis posteriorly.

Within Clade 1 there are four moderately distinct morphotypes with more subtle distinctions enabling the marginal recognition of a fifth. In Clade 2, there are two clearly distinct morphotypes, one of which divides imperfectly on the basis of the anterior extent of the vitelline follicles. In Clade 3 there are two clearly distinguishable morphotypes corresponding to the two previously described species, *B. neglecta* and *B. unexpecta*.

### Synthesis

An integrative approach to the delineation of trematode species was adopted, considering morphology, molecular data and biology (principally host-specificity), following the species recognition criteria proposed by Bray *et al*. (2022). These criteria require reciprocal monophyly inferred from at least the most informative molecular marker plus either morphological distinction or biological distinction in the form of differing host distributions. Application of these criteria leads us to recognize 10 species in the collection of bivesiculids from holocentrids, muraenids and serranids. Recognition of almost all of these species can be considered difficult in that the morphological differences between them are limited. This hypothesis requires the recognition of several combinations of actually or nearly cryptic species. Here the broad overall case for the recognition of 10 species (nine named) is made, because the argument requires reference to multiple species simultaneously.

One species, *Bivesicula nana* n.sp., has no molecular data but is arguably the most morphologically distinctive of those considered here on the basis of being far smaller than all the other morphologically comparable species. On the basis of its overall form, it can be predicted that it will prove to belong to Clade 1.

The nine sequenced species are all easily distinguished by *cox*1 data but far less so, or in some cases not at all, by ITS2 rDNA and 28S rDNA data. The two ribosomal markers are effective in distinguishing the three major clades of *Bivesicula* species and the three species of Clade 2 recognized, but are completely ineffectual in delineating the species of Clade 1 suggested by *cox*1 data. In this context, it is noteworthy that the two previously described Clade 3 species for which new sequence data are reported (*B. neglecta* and *B. unexpecta*), are morphologically clearly distinct and infect a non-overlapping range of hosts (apogonids and a pomacentrid, respectively) yet differ at only a single base position in the ITS2 rDNA alignment and three base positions in the 28S rDNA alignment. Thus, as found recently for certain Aporocotylidae (Cutmore *et al*., 2021), ITS2 rDNA and 28S rDNA sequences are uninformative for the distinction of some combinations of bivesiculid species. The considerations below are thus based on inferences drawn from morphology, *cox*1 sequences and biology (host-specificity). Reference to Figs 2 and 3 demonstrates that some of the species discussed below (especially those of Clade 2) are distinguished by ITS2 rDNA and 28S rDNA sequences, but the taxonomic hypothesis does not depend on those data.

Clade 1 here is interpreted as representing seven species if the morphologically comparable but unsequenced *B. nana* n.sp. is included. Of the six sequenced putative species, relating to nine OTUs, the form from *E. fasciatus* from Bali is represented by a single highly distinctive sequence (differing from all other taxa at a minimum of 50 base positions in the *cox*1 alignment); however, there is no morphological specimen available, and this form is consequently only noted here as an indication of uncharacterized richness. The five remaining species are morphologically similar and incorporate substantial difficulty in delineation, although they differ from each other at a minimum of 21 *cox*1 base positions. Three distinguishable morphotypes were detected, relating to that of *B. claviformis*, *B. cephalopholicola* n.sp. and *B. obovata* Shimazu and Machida, 1995. Of these, *B. claviformis* is considered actually or nearly morphologically cryptic with respect to two further species, *B. sheni* n.sp. and *B. polynesiensis* n.sp.

Samples from serranids from Okinawa are here interpreted as representative of *B. claviformis*, the type species for the genus. These specimens form a well-supported *cox*1 clade with samples from serranids from Ningaloo Reef (Western Australia) and from holocentrids and serranids from the GBR (Queensland). They differ at a maximum of 23 base positions in the *cox*1 alignment and relate to OTUs 4, 5, 6 and 7; these differences are here interpreted as reflecting intra-specific geographical populations. The entire clade differs from all other taxa in the analysis at a minimum of 37 base positions. The morphology of specimens corresponding to the sequenced specimens is broadly uniform, but does incorporate variation in body size, shape, the appearance of the vitelline follicles and their anterior extent. This is the only species represented in Clade 1 in which the *cox*1 OTUs do not correspond directly to just one of the recognized species.

*Bivesicula sheni* n.sp. is described as a new species explicitly cryptic relative to *B. claviformis* and presently known only from the GBR (at both Heron Is. and Lizard Is.). It has been collected and sequenced from two species of *Sargocentron* Fowler (Holocentridae) and six species of *Epinephelus* Bloch (Serranidae). Twenty *cox*1 sequences differ at only 0–4 base positions. As for *B. claviformis*, the morphology of specimens corresponding to the sequenced specimens is broadly uniform, but again incorporates noticeable variation in body size, shape, the appearance of the vitelline follicles and their anterior extent. There is also evidence of host-induced phenotypic variability in this species relating to infection of holocentrids and serranids. No basis for the morphological distinction of this species from *B. claviformis*, which occurs in an overlapping range of fishes on the GBR, was identified; indeed, the two species have been sequenced from the same individual *Sargocentron caudimaculatum* (Rüppell). However, in the *cox*1 analysis, this species differs from those identified as *B. claviformis* at 39–46 base positions. In addition, samples identified as *B. sheni* n.sp. form a well-supported clade with samples identified as the previously described species *B. obovata. Bivesicula obovata* is morphologically clearly distinguishable from both *B. claviformis* and *B. sheni* n.sp. on the basis of its massive body form. The criteria for the recognition of species proposed by Bray *et al*. (2022) constrain us to recognize *B. sheni* n.sp. as a species separate from *B. claviformis* because, despite indistinguishable morphology and overlapping distribution and hosts, the topology of their relationships based on *cox*1 data does not identify them as sister taxa, but as separated by another clearly distinct species. On this basis, *B. claviformis* and *B. sheni* n.sp. can presently be distinguished where they co-occur only by sequence data (*cox*1). Therefore, the designated holotype for the new species is a hologenophore and the paratypes are all hologenophores and paragenophores.

*Bivesicula polynesiensis* n.sp. is proposed for samples from two species of Holocentridae and two of Serranidae from the Austral, Gambier and Society Archipelagos in French Polynesia. Corresponding sequences form a strongly supported *cox*1 clade with a minimum of 38 base positions differences relative to all other taxa. There is no population structure between specimens from the three French Polynesian collecting localities which are separated by up to 1600 km. This species closely resembles *B. claviformis* and *B. sheni* n.sp. to the point of being effectively cryptic, however it does not form a monophyletic clade with either species. Relationships between *B. cephalopholicola* n.sp., *B. claviformis, B.sheni* n.sp., *B. obovata* and *B. polynesiensis* n.sp. are poorly resolved overall, but as for the recognition of *B. sheni* n.sp., the close relationship between *B. sheni* n.sp. and *B. obovata* precludes recognition of *B. polynesiensis* n.sp. as forming a clade with *B. claviformis* or *B. sheni* n.sp. Slight tendencies to morphological distinction of *B. polynesiensis* n.sp. relative to *B. claviformis* and *B. sheni* n.sp. are discussed following the species account, but the three are really very similar. Complicating morphological differentiation is the fact that, as for *B. sheni* n.sp., there was clear evidence of host-induced phenotypic variation between samples from holocentrids and serranids. Strikingly, the variation (a tendency to a more rounded body in specimens from holocentrids than in those from serranids) is comparable to that seen for *B. sheni* n.sp. so that what can be interpreted as different species have convergent morphology depending on the family of hosts from which they are recovered. On present indications, this species can probably be identified reliably on the basis of its *B. claviformis*-like morphology in the context of infection of holocentrids and serranids in French Polynesia. However, it is clear that molecular data are necessary for identification in any other circumstances. To minimize any ambiguity associated with the description of *B. polynesiensis* n.sp., it is described with a hologenophore as the holotype and hologenophores and paragenophores as the paratypes.

*Bivesicula cephalopholicola* n.sp. is proposed for specimens from three species of *Cephalopholis* Bloch & Schneider from Lizard Is. This form is distinct from all other taxa at a minimum of 44 *cox*1 base positions. The specimens broadly resemble *B. claviformis*, *B. sheni* n.sp., *B. nana* n.sp. and *B. polynesiensis* n.sp., but they show some morphological distinction in their relatively small cirrus-sacs, biologically in their restriction to species of the genus *Cephalopholis* and, on the GBR, occurring only at Lizard Is., not at Heron Is. Neither *B. claviformis* nor *B. sheni* n.sp. has been detected in species of *Cephalopholis* at Lizard Is. although one infection of *B. claviformis* was found in *C. argus* at Okinawa. Specimens from *Cephalopholis boenak* (Bloch) from New Caledonia are identified as this species on the basis of their morphology and host distribution.

Clade 2 is here interpreted as comprising three species, *Bivesicula gymnothoracis* Shimazu and Machida, 1995, *Bivesicula palauensis* Shimazu and Machida, 1995 and *Bivesicula novaecaledoniensis* n.sp. The two previously described species are readily morphologically distinguishable by general body features. New specimens identified as *B. palauensis* were collected from *Variola albimarginatus* Baissac and *Epinephelus areolatus* (Forsskål) from Okinawa (with molecular data) and from *Epinephelus morrhua* (Valenciennes) from New Caledonia (morphology only). These samples are consistent with the original (and only previous) description of this species from the Philippines (Shimazu and Machida, 1995). Specimens identified as *B. gymnothoracis* were recollected from the type host, *Gymnothorax kidako* (Muraenidae), from Minabe, Japan and also, surprisingly, from the completely unrelated *E. fasciatus* (Serranidae) from Minabe and Okinawa. New specimens from *G. kidako* are far larger than almost all those from *E. fasciatus* but whether this relates to a host-induced morphological distinction, or the limited sample size is not known. Specimens from *G. kidako* and *E. fasciatus* from Minabe (OTU 13) incorporated variation at only 0–4 base positions in the *cox*1 alignment; samples from *E. fasciatus* from Okinawa (OTU 12) differed from the Minabe samples at 10–13 base positions in the *cox*1 dataset, a distinction interpreted as intraspecific geographical variation. Specimens from *Epinephelus chlorostigma* (Valenciennes) from New Caledonia are here described as a new species. This species resembles *B. gymnothoracis* closely, differing unreliably in only one detected morphological character, but the two species differ at 81–85 base positions in the *cox*1 alignment, 8–9 base positions in the ITS2 rDNA alignment, 13 in the 28S rDNA alignment, and are not sister taxa.

Table 1 summarizes the geographic distribution of the 10 species on the basis of the specimens reported here. Notably, four of the 10 have been found from off Lizard Island, northern GBR. Table 2 summarize the host distribution. Notably, seven of the species have been found in *E. fasciatus.* All 10 species infect serranids; four also infect holocentrids and one also infects a muraenid.

**Table 2.** Host distribution of 10 species of *Bivesicula* collected in Indo-west Pacific localities

Host	1	2	3	4	5	6	7	8	9	10
H: *Neoniphon sammara*								⬤		
H: *Sargocentron caudimaculatum*		⬤						⬤	⬤	
H: *Sargocentron diadema*								⬤		
H: *Sargocentron microstoma*								⬤		
H: *Sargocentron rubrum*						⬤			⬤	
H: *Sargocentron spiniferum*		⬤						⬤	⬤	
M: *Gymnothorax kidako*			⬤							
S: *Cephalopholis argus*		⬤								
S: *Cephalopholis boenak*	⬤									
S: *Cephalopholis cyanostigma*	⬤									
S: *Cephalopholis microprion*	⬤									
S: *Cephalopholis urodeta*								⬤		
S: *Epinephelus areolatus*							⬤			
S: *Epinephelus chlorostigma*					⬤					
S: *Epinephelus cyanopodus*									⬤	
S: *Epinephelus fasciatus*		⬤	⬤		⬤	⬤		⬤	⬤	⬤
S: *Epinephelus maculatus*				⬤					⬤	
S: *Epinephelus merra*		⬤							⬤	
S: *Epinephelus morrhua*							⬤			
S: *Epinephelus ongus*									⬤	
S: *Epinephelus quoyanus*						⬤			⬤	
S: *Epinephelus tauvina*		⬤								
S: *Epinephelus undulatostriatus*						⬤			⬤	
S: *Variola albimarginata*							⬤			

H, Holocentridae; M, Muraenidae; S, Serranidae.

1. *B. cephalopholicola* n.sp.; 2.*B. claviformis*; 3.*B. gymnothoracis*; 4.*B. nana* n.sp.; 5.*B. novaecaledoniensis* n.sp.; 6.*B. obovata*; 7.*B. palauensis*; 8.*B. polynesiensis* n.sp.; 9.*B. sheni*; 10. *B.* ‘Bali'sp.

#### Taxonomy

***Bivesicula*** Yamaguti, 1934

***Bivesicula* Clade 1**

**Fig. 4.**
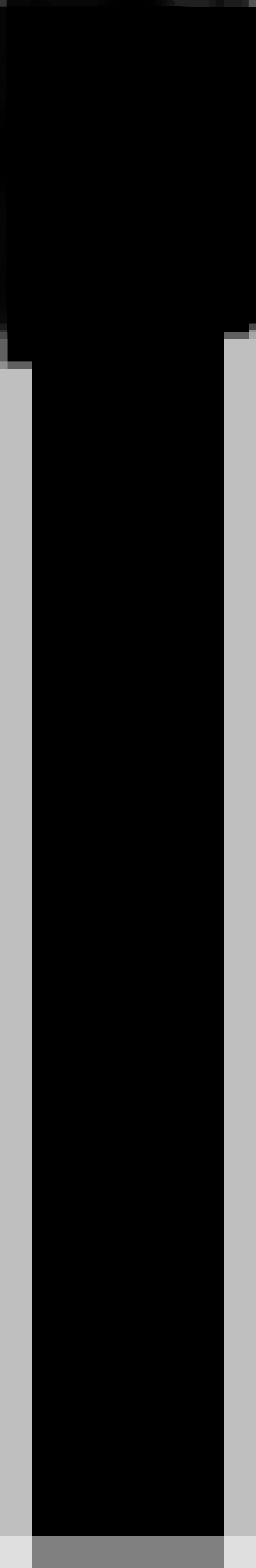
*Bivesicula claviformis* Yamaguti, 1934. (A) from *Epinephelus fasciatus,* Okinawa, (B) from *Cephalopholis argas*, Okinawa, (hologenophore, *cox*1 OTU 4), (C) from *Epinephelus merra* Okinawa, (hologenophore, *cox*1 OTU 4), (D) from *E. merra,* Lizard Is., GBR, (paragenophore, *cox*1 OTU 7), (E) from *Sargocentron spiniferum,* Lizard Is. (paragenophore, *cox*1 OTU 7), (F) from *E. merra* Lizard Is., (paragenophore, *cox*1 OTU 7), (G) from *E. fasciatus,* Ningaloo Reef, (H) from *E. fasciatus,* Ningaloo Reef (paragenophore, *cox*1 OTU 5), (I) from *E. fasciatus,* Ningaloo Reef (paragenophore, *cox*1 OTU 6). Scale bars = 200 *μ*m; all images to same scale.

***Bivesicula claviformis*** Yamaguti, 1934 (Fig. 4)

Syn. *Bivesicula epinepheli* Yamaguti, 1938

*Bivesicula xishaensis* Gu and Shen, 1983

Type-host: *Seriola quinqueradiata* Temminck & Schlegel, Japanese amberjack (Carangidae).

Type-locality: Inland Sea, Japan.

Other records: see Remarks

#### This study

Hosts: Serranidae – *Cephalopholis argus* Schneider, Peacock hind; *Epinephelus fasciatus* (Forsskål), Blacktip grouper; *Epinephelus merra* Bloch, Honeycomb grouper; *Epinephelus tauvina* (Forsskål), Greasy grouper. Holocentridae – *Sargocentron caudimaculatum* (Rüppell), Silverspot squirrelfish; *Sargocentron spiniferum* (Forsskål), Sabre squirrelfish.

Localities: Okinawa, Japan (Tomari Fish Market); Ningaloo Reef (WA); Lizard Is., northern GBR.

Site in host: Intestine and pyloric caeca.

Prevalences: Serranidae – *C. argus* 1 of 1 Okinawa, Japan; *E. fasciatus* 3 of 5 Okinawa, Japan; 5 of 8 Ningaloo Reef; *E. merra* 1 of 20 Lizard Is.; 1 of 3 Okinawa, Japan; *E. tauvina* 1 of 3 Ningaloo Reef. Holocentridae – *S. caudimaculatum* 1 of 6 Lizard Is.; *S. spiniferum* 1 of 21 Lizard Is. Prevalences for Lizard Is. may be underestimates because they are based on sequenced worms only.

Deposited material: MPM six vouchers from Japan, 21840–2; WAM 16 vouchers from Ningaloo Reef, V10949–64; 13 vouchers from Lizard Is., QM G239556–68.

Representative DNA sequences: Partial *cox*1 mtDNA, 10 sequences (all submitted to GenBank, OM456619–28); ITS2 rDNA, 11 sequences (six submitted to GenBank, OM523327–32); partial 28S rDNA, four sequences (all submitted to GenBank, OM459980–83).

ZooBank LSID: urn:lsid:zoobank.org:act:27EB5145-4671-45AE-A0CD-558355110A88.

*Description*. 32 specimens examined; description based on all new material listed above. GBR specimens are exclusively hologenophores or paragenophores. Measurements below are for the 23 entire specimens. Measurements for five individual host/locality combinations are given in Supplementary Table 1.

Body relatively elongate, fusiform, 739–1539 × 312–567 (1143 × 440); length/width ratio 1.82–3.46 (2.55). Tegument with minute spines, most obvious anteriorly. Eyespot pigment dispersed widely at level of oesophagus. Prepharynx short. Pharynx robust, usually as wide as long, 72–161 × 90–157 (120 × 125); length/width 0.75–1.36 (0.96). Oesophagus sinuous, 73–163 (114) long. Caeca blind, of equal length, reaching to 191–450 (312), or 21.1–31.8 (27.2) % body length, from posterior extremity of body. Testis single, entire, post-equatorial, at level of ends of intestinal caeca, 90–220 × 84–204 (151 × 140), 518–1067 (764), or 62.5–73.2 (67.7) % body length, from anterior extremity. External seminal vesicle rounded, sometimes extending clearly anterior to cirrus-sac, often entirely dorsal to cirrus-sac. Cirrus-sac median, orientated antero-posteriorly, filled with prostatic cells, 138–309 × 87–186 (232 × 131), 347–770 (510), or 40.3–52.2 (44.8) % body length, from anterior extremity. Internal seminal vesicle entire. Pars prostatica diverticulate and complex. Genital pore median, close to level of posterior margin of cirrus-sac. Ovary subspherical, unlobed, dorso–dextral to cirrus-sac, 44–130 × 44–113 (85 × 74), 233–568 (395), or 30.1–40.2 (34.3) % body length, from posterior extremity. Uterine seminal receptacle frequently visible. Vitelline follicles variable in size and density, distributed from 136 to 251 (189), or 13.4–21.3 (16.7) % body length, from anterior extremity, usually almost at level of anterior arms of excretory vesicle but sometimes leaving tips clearly exposed, to 219–508 (370), or 25.4–36.5 (32.2) % body length, from posterior end of body, usually terminating distinctly anterior to ends of intestinal caeca but sometimes extending almost to their termination; total vitelline field 367–815 (584) long or 45.2–56.3 (51.2) % of body length. Uterus passes to close to posterior extremity before passing anteriorly to open at common genital pore. Eggs 68–90 × 35–49 (44 × 77). Excretory vesicle V-shaped; arms pass latero-dorsally to testis, terminating distinctly posterior to posterior margin of pharynx, 124–205 (161), or 11.5–18.3 (14.4) % body length, from anterior extremity. Excretory pore terminal.

#### Remarks

This species is the type species for the genus and, as is commonly the case, it is by far the most frequently reported species in the genus. There remain some problems in the understanding of the status of this species. The type-host was reported as *Seriola quinqueradiata* Temminck & Schlegel (Carangidae) from the Inland Sea of Japan (Yamaguti, 1934). Apparently, no carangid has been reported as a host for a bivesiculid since. Thus, if the species is actually one that shows high specificity for *S. quinqueradiata*, then it may be that the species has not been re-reported since its first description; however, that seems unlikely and probably the original infection was exceptional. Yamaguti (1938) described a second species, *B. epinepheli* Yamaguti, 1938, from *Epinephelus akaara* (Temminck & Schlegel), also from the Inland Sea. Although Yamaguti (1938) stated that this species differs notably from *B. claviformis* in the position of the pharynx, the differences appear inconsequential and the two species were synonymized by Cribb *et al*. (1994), an action accepted by Shimazu (1994) and Shimazu and Machida (1995). *Bivesicula xishaensis* Gu and Shen, 1983 was described on the basis of a single specimen from *Epinephelus fasciatus* collected in the Xisha Islands, off China (Gu and Shen, 1983). The description lacks some important details (e.g. the anterior extent of the excretory vesicle) but generally resembles *B. claviformis* and it was synonymized with that species by Cribb *et al*. (1994).

The specimens (hologenophores and paragenophores) corresponding to sequences generated in this study from *C. argus*, *E. fasciatus* and *E. merra* from Okinawa are broadly consistent with the original descriptions of *B. claviformis* (and *B. epinepheli*) from the same area. More recent records of *B. claviformis* by Shimazu and Machida (1995) and Kuramochi (2018) from Japanese waters have been overwhelmingly from serranids so that, despite the issue with the identity of the type host for *B. claviformis*, the molecular identity of this species can probably now be considered established with reasonable confidence. The *cox*1 sequences from Okinawan serranids form a strongly supported clade with sequences from samples from *E. fasciatus* from Ningaloo Reef and from *E. merra*, *S. caudimaculatum* and *S. spiniferum* from Lizard Is. Variation between geographic populations of this clade are at 17–23 base positions in the *cox*1 dataset, a level consistent with what has been interpreted as intra-specific geographic variation for other trematodes such as species of *Hurleytrematoides* (see McNamara *et al*., 2014) and *Preptetos* (see Bray *et al*., 2022). Differences between these localities in the ITS2 and 28S rDNA alignments are no greater than 1 and 2 base positions, respectively.

The specimens of this species are by no means identical; there is variation in body shape, size of the vitelline follicles and appearance of the pharynx. However, no regional pattern of variation was detected (variation in specimens from a single locality was as great as that between localities) and the specimens are broadly consistent with each other. All these forms are thus interpreted as *B. claviformis*.

The evidence discussed above suggests that *B. claviformis* occurs widely in the Indo-Pacific. However, the molecular analyses also show the presence of two other strongly supported independent *cox*1 clades from French Polynesia and the GBR, respectively, for which the corresponding specimens have highly similar morphology. Given the topology of the relationships between the various clades of species of *Bivesicula*, those clades are recognized as representing distinct species. The description of those species calls into question the reports of *B. claviformis* as being widespread across the IWP. Reports from China (Gu and Shen, 1983), the Red Sea (Nagaty, 1948), Fiji (Manter, 1961; Rigby *et al*., 1997; Nahhas *et al*., 2004) and Indonesia (Fischthal and Kuntz, 1965) all require genetic characterization and further morphological study to explore their status. It is unlikely to be a coincidence that the area most heavily sequenced for specimens resembling *B. claviformis*, the GBR, is that for which there is evidence of sympatric cryptic species. It is thus possible, perhaps probable, that sympatric cryptic species will also be found elsewhere.

**Fig. 5.**
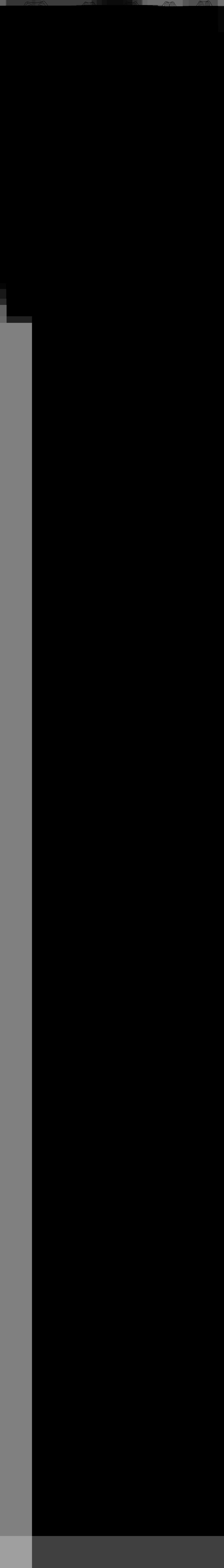
*Bivesicula sheni* n.sp. (A) Holotype (hologenophore, *cox*1 OTU 1) from *Epinephelus fasciatus* from Lizard Is., (B) from *E. fasciatus*, Lizard Is. (paragenophore, *cox*1 OTU 1), (C) from *E. fasciatus*, Lizard Is. (paragenophore, *cox*1 OTU 1), (D) from *Epinephelus undulatostriatus* from Lizard Is. (paragenophore, *cox*1 OTU 1), (E) from *E. quoyanus* from Lizard Is., (F) from *E. merra* from Lizard Is., (G) from *Sargocentron spiniferum* from Lizard Is., (hologenophore, *cox*1 OTU 1), (H, I) from *S. spiniferum* from Lizard Is., (paragenophores, *cox*1 OTU 1). Scale bars = 200 *μ*m.

***Bivesicula sheni*** n.sp. (Fig. 5)

Type-host: *Epinephelus fasciatus* (Forsskål), Blacktip grouper (Serranidae).

Type-locality: Lizard Is., northern GBR.

Other hosts: Serranidae – *Epinephelus cyanopodus* (Richardson), Speckled blue grouper; *Epinephelus maculatus* (Bloch), Highfin grouper; *Epinephelus merra* Bloch, Honeycomb grouper; *Epinephelus ongus* (Bloch), White-streaked grouper; *Epinephelus quoyanus* (Valenciennes), Longfin grouper; *Epinephelus undulatostriatus* (Peters), Maori rockcod. Holocentridae – *Sargocentron caudimaculatum* (Rüppell), Silverspot squirrelfish; *Sargocentron rubrum* (Forsskål), Redcoat; *Sargocentron spiniferum* (Forsskål), Sabre squirrelfish.

Other localities: Heron Is., southern GBR.

Site in host: Intestine and pyloric caeca.

Prevalences: (listed as confirmed *cox*1 sequence/additional morphology only/total examined). Holocentridae – *S. rubrum* 0/1/27 Heron Is.; *S. caudimaculatum* 2/2/6 Lizard Is.; *S. spiniferum* 4/5/21 Lizard Is. Serranidae – *E. cyanopodus* 0/1/9 Heron Is., 2/1/4 Lizard Is.; *E. fasciatus* 3/6/63 Heron Is., 2/1/4 Lizard Is.; *E. maculatus* 1/3/10 Lizard Is.; *E. merra* 2/3/20 Lizard Is.; *E. ongus* 0/1/3 Lizard Is.; *E. quoyanus* 1/2/74 Heron Is., 2/4/20 Lizard Is.; *E. undulatostriatus* 1/1/4 Heron Is.

Deposited material: QM Holotype, G239569; 34 paratypes, G239570–603; 58 vouchers, G239604–62; NHMUK 20 paratypes, 2022.2.15.14–33, 32 vouchers, 2022.2.15.34–65.

Representative DNA sequences: Partial *cox*1 mtDNA, 20 sequences (16 submitted to GenBank, OM456662–77); ITS2 rDNA, eight sequences (seven submitted to GenBank, OM523347–53); partial 28S rDNA, three sequences (all submitted to GenBank, OM459995–97).

ZooBank LSID: urn:lsid:zoobank.org:act:0BD486A1-B1F3-44E3-AE89-0A71D5BB1550.

Etymology: This species is named after Dr Jiwei Shen in recognition of his major contribution to knowledge of trematodes of fishes of our region.

*Description*. 157 specimens examined; description based on all examined material. Specimens are hologenophores, paragenophores and samples inferred to be this species. Measurements given below are for 30 entire measured specimens, all paragenophores. Measurements for individual host/locality combinations are given in Supplementary Table 2.

Body shape variable, from relatively elongate, with almost parallel sides to distinctly fusiform, 827–1640 × 342–657 (470 × 1237); length/width ratio 1.82–3.56 (2.65). Tegument with minute spines, most obvious anteriorly. Eyespot pigment dispersed widely at level of oesophagus. Prepharynx short. Pharynx robust, 75–167 × 93–174 (133 × 130), highly variable in shape; length/width ratio 0.71–1.41 (0.97). Oesophagus sinuous, 61–191 (134) long, often heavily obscured by vitelline follicles. Caeca blind, of equal length, reaching to 183–453 (303), or 20.3–32.9 (24.5) % body length, from posterior extremity of body. Testis single, entire, post-equatorial, at level of ends of intestinal caeca, 82–223 × 78–205 (176 × 158), 575–1198 (864), or 65.0–75.1 (70.0) % body length, from anterior extremity. External seminal vesicle rounded, sometimes extending clearly anterior to cirrus-sac, often entirely dorsal to cirrus-sac. Cirrus-sac median, orientated antero-posteriorly, filled with prostatic cells, 180–322 × 105–204 (260 × 151), 378–847 (578), or 41.4–52.9 (46.7) % body length, from anterior extremity. Internal seminal vesicle entire. Pars prostatica diverticulate and complex. Genital pore median, close to level of posterior margin of cirrus-sac. Ovary subspherical, unlobed, dorso-dextral to cirrus-sac, 67–132 × 62–123 (100 × 86) perpendicular width, 251–513 (393), or 25.5–37.1 (31.7) % body length, from posterior extremity. Uterine seminal receptacle frequently visible. Vitelline follicles strikingly variable in size and density, distributed from 125 to 301 (209), or 13.2–22.5 (17.0) % body length, from anterior extremity, typically almost at level of anterior arms of excretory vesicle and frequently leaving tips clearly exposed, to 248–488 (383), or 26.5–36.4 (30.9) % body length, from posterior end of body, usually terminating distinctly anterior to ends of intestinal caeca but sometimes extending to their termination; total vitelline field 428–890 (644) long or 26.5–36.4 (30.9) % of body length. Uterus passes to close to posterior extremity before passing anteriorly to open at common genital pore. Eggs 64–87 × 32–52 (75 × 42). Excretory vesicle V-shaped; arms pass latero-dorsally to testis, terminating distinctly posterior to posterior margin of pharynx, 127–223 (167), or 9.9–20.6 (13.8) % body length, from anterior extremity. Excretory pore terminal.

#### Remarks

Because of the difficulty of distinguishing this species from *B. claviformis* (see below), the species is based entirely on type specimens that are hologenophores and paragenophores. Measurements given above all relate to paragenophores. However, in the prevalence listing above, unsequenced specimens have been tentatively identified as *B. sheni* n.sp. to give an indication of the overall prevalence detected. The specimens are tentatively listed as *B. sheni* n.sp. on the basis that overall sequencing shows that species to be far more common in GBR fishes than is *B. claviformis*, which has only been found so far in the northern GBR. Notably, three of the 13 host/locality combinations (*E. cyanopodus* and *S. rubrum* from Heron Is. and *E. ongus* from Lizard Is.) have never been sequenced. Supplementary Table 2 lists measurements of 96 adult whole-mount specimens that are tentatively assigned to this species; the 96 includes 30 paragenophore specimens.

**Fig. 6.**
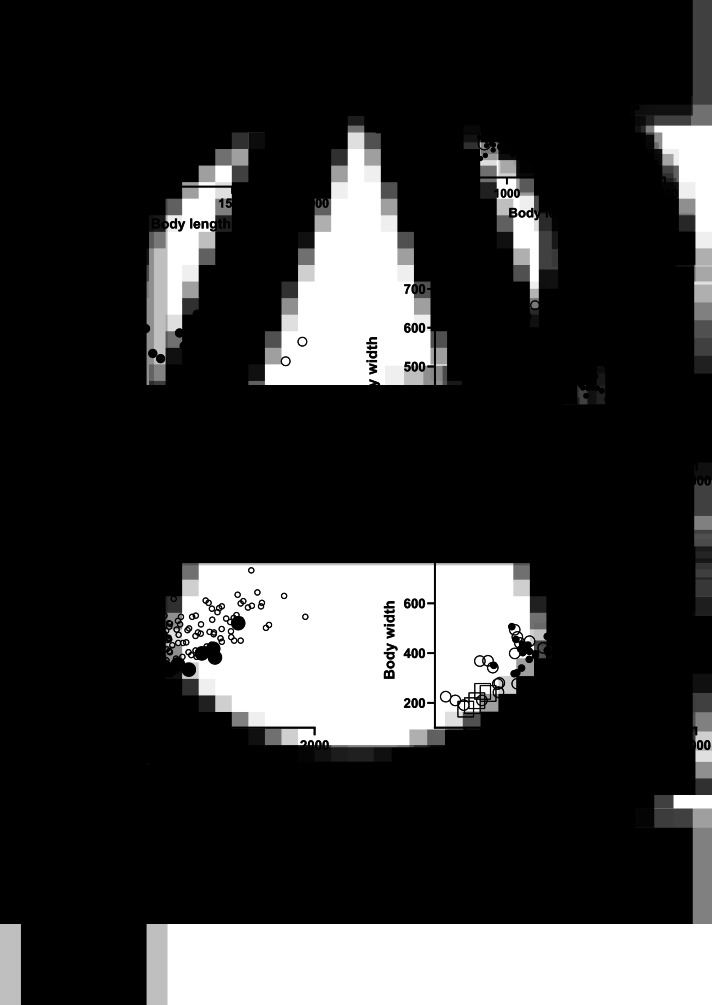
Morphometric comparisons of *Bivesicula* species. (A) Paragenophores of *Bivesicula sheni* n.sp. from GBR Holocentridae (○) and Serranidae (⬤), (B) *Bivesicula obovata* from original description from *Sargocentron rubrum* from Japan (Δ), from *S. rubrum* from the Great Barrier Reef (□) relative to *B. sheni* n.sp. from the GBR (⬤) including one from *S. rubrum* (○), (C) *Bivesicula polynesiensis* n.sp. from French Polynesian Holocentridae (⬤) and Serranidae (○). (D) *B. polynesiensis* n.sp. from Holocentridae (○) and Serranidae (▴) relative to *B. sheni* n.sp. from Holocentridae (□) and Serranidae (⬤), (E) *Bivesicula cephalophololicola* n.sp. (⬤) relative to combined *B. claviformis, B.polynesiensis* n.sp. and *B. sheni* n.sp. (○), (F) Gravid *Bivesicula nana* n.sp. (□) relative to immature (○) and gravid (⬤) *B. sheni* n.sp.

Figure 6A shows the distribution of body length relative to width for specimens interpreted as *B. sheni* n.sp. distinguished by host family (Holocentridae or Serranidae); although there is overlap in body shape of specimens from the two fish families, there is a clear tendency for specimens from holocentrids to be broader than those from serranids. In addition, 67 of 80 specimens measured from serranids are longer than the longest of 16 specimens from holocentrids suggesting strongly that total size is also affected by host identity. It was thus a surprise when sequence data indicated that samples from GBR holocentrids and serranids were the same species; their appearance at the time of collection together with preconceptions with respect to host-specificity led to an expectation that they represented different species.

In the combination of the intestinal caeca not extending posterior to the testis, the vitelline follicles not reaching the posterior margin of the pharynx or extending significantly anterior to the excretory vesicle, and the pharynx being larger than the eggs, this species is clearly distinct from all existing species of *Bivesicula* except for *B. claviformis* and *B. lutiani.* Of these, *Bivesicula lutiani* is a problematic species. It was described on the basis of 23 specimens (a robust sample) from one of six *Lutjanus kasmira* examined from off the Xisha Islands, Japan by Gu and Shen (1983). The host record is intriguing because species of *Lutjanus*, including *L. kasmira*, have been heavily studied for their digenean parasites without other reports of species of *Bivesicula*. Thus, the infection is either atypical (a species normally infecting another fish species) or exceptional (genuine, but isolated). Gu and Shen (1983) observed that the species closely resembles *B. claviformis* Yamaguti, 1934 and *B. epinepheli* Yamaguti, 1934 (now a synonym of *B. claviformis*). It was distinguished from the former by the size of body, the width and position of the cirrus sac, and the position of the testes, and from the latter in the shape and the size of the body, the position of ovary and the size of the eggs. In light of the level of variation seen in other species of *Bivesicula*, it seems likely that none of these characters reliably delineates *B. lutiani.* The status of this species requires further attention.

The key comparison for *B. sheni* n.sp. is relative to the type species, *B. claviformis*. Samples here identified as *B. sheni* n.sp. differ from those of *B. claviformis* (reported above) at 39–45 base positions in the *cox*1 dataset. Both taxa form strongly supported clades. Most significantly, in terms of the recognition of *B. sheni* n.sp. as a new species, is the observation that its closest relative in analysis of *cox*1 sequences is *B. obovata* (characterized below), from which it differs clearly in morphology and with which it forms a strongly supported clade. *Bivesicula sheni* n.sp. and *B. obovata* infect an overlapping range of holocentrid and serranid fishes. When ITS2 and 28S rDNA sequences are considered, there are almost no differences between *B. claviformis*, *B. sheni* n.sp. and *B. obovata*. Corresponding ITS2 rDNA sequences may be identical between the three and never differ by more than 2 base positions. Corresponding 28S rDNA sequences differ between *B. claviformis* and the other two forms at 2–4 base positions and between *B. sheni* n.sp. and *B. obovata* at 0–2 base positions. None of the differences for either marker results in the recognition of clades distinguishing any of these forms which occur sympatrically on the GBR. In short, *B. claviformis* and *B. sheni* n.sp. are morphologically indistinguishable, a problem exacerbated by the considerable intraspecific variation in both, including host-induced morphological variation (dependent on host family) for *B. sheni* n.sp. Charting of numerous morphometric characters for the two forms failed to detect any reliable basis for distinction. The vitelline follicles of *B. claviformis* tend to be slightly more anteriorly extensive than those of *B. sheni* n.sp., but the distinction is not reliable. *Bivesicula sheni* n.sp. is thus here recognized explicitly as a species cryptic relative to *B. claviformis* and is distinguished on the basis of consistent genetic differences (principally in *cox*1) and the fact that it does not form a clade with *B. claviformis*.

**Fig. 7.**
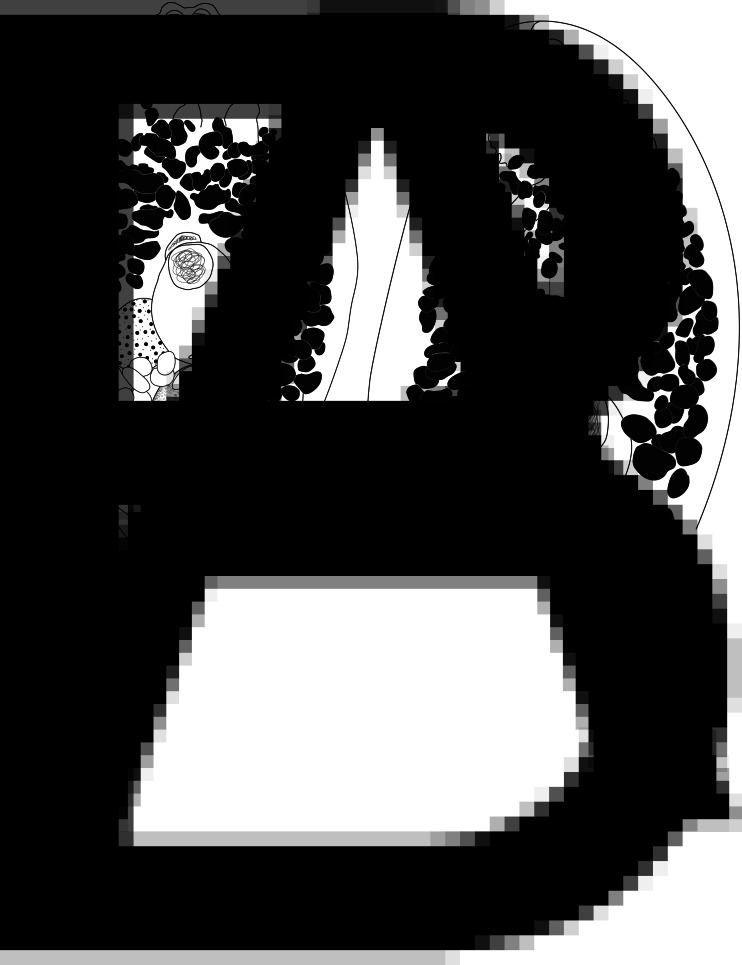
*Bivesicula obovata* Shimazu and Machida, 1995. (A) From *Sargocentron rubrum* from Heron Is. (paragenophore, *cox*1 OTU 2), (B) from *Epinephelus quoyanus* from Heron Is. (hologenophore, *cox*1 OTU 2). Scale bars = 200 *μ*m.

***Bivesicula obovata*** Shimazu and Machida, 1995 (Fig. 7)

Type host: *Sargocentron rubrum* (Forsskål), Redcoat (Holocentridae).

Type locality: Nago, Okinawa Prefecture, Japan.

#### This study

Hosts: Holocentridae – *S. rubrum*. Serranidae – *Epinephelus fasciatus* (Forsskål), Blacktip grouper; *Epinephelus quoyanus* (Valenciennes), Longfin grouper; *Epinephelus undulatostriatus* (Peters), Maori rockcod.

Localities: Heron Is., southern GBR.

Site in host: Intestine.

Prevalences: Holocentridae – *S. rubrum* 1 of 27. Serranidae – *E. quoyanus* 1 of 74; *E. undulatostriatus* 1 of 3; *E. fasciatus* 1 of 63.

Deposited material: QM four vouchers, G239663–6.

Representative DNA sequences: Partial *cox*1 mtDNA, four sequences (all submitted to GenBank, OM456640–43); ITS2 rDNA, two sequences (both submitted to GenBank, OM523338–39); partial 28S rDNA, two sequences (both submitted to GenBank, OM459988–89).

ZooBank LSID: urn:lsid:zoobank.org:act:6D9EA2DA-F6B9-407E-8F69-87816CA0C673.

*Description*. Four specimens examined; measurements are of two paragenophore specimens from *S. rubrum*, Heron Is.

Body relatively massive, fusiform, 1285–1379 × 819–829. Length/width ratio 1.57–1.66. Tegument with minute spines, most obvious anteriorly. Eyespot pigment dispersed widely at level of oesophagus. Prepharynx short. Pharynx robust, approximately as wide as long, 136 × 132–137; length/width ratio 0.99–1.03. Oesophagus sinuous, 122–196 long. Caeca blind, of equal length, reaching to 320–389 or 24.9–28.2% body length, from posterior extremity of body. Testis single, entire, post-equatorial, at level of ends of intestinal caeca, 250–266 × 247–262, 849–860 or 62.4–66.1% body length, from anterior extremity. External seminal vesicle rounded, sometimes extending clearly anterior to cirrus-sac, often entirely dorsal to cirrus-sac. Cirrus-sac median, orientated antero-posteriorly, filled with prostatic cells, 279–340 × 205–230, 523–559 or 37.9–43.5% body length, from anterior extremity. Internal seminal vesicle entire. Pars prostatica diverticulate and complex. Genital pore median, close to level of posterior margin of cirrus-sac. Ovary subspherical, unlobed, dorso-dextral to cirrus-sac, 178–208 × 177–201, 432–512 or 33.6–37.1% body length, from posterior extremity. Uterine seminal receptacle frequently visible. Vitelline follicles distributed from 218–272 or 17.0–19.7% body length, from anterior extremity, close to level of anterior arms of excretory vesicle but leaving tips clearly exposed, to 350–440 or 27.2–31.9% body length, from posterior end of body, terminating distinctly anterior to ends of intestinal caeca; total vitelline field 667–717 long or 48.4–55.8% of body length. Uterus passes to close to posterior extremity before passing anteriorly to open at common genital pore. Eggs 71–80 × 41–49 (77 × 45). Excretory vesicle V-shaped; arms pass latero-dorsally to testis, terminating close to posterior margin of pharynx, 194–204 or 14.8–15.1% body length, from anterior extremity. Excretory pore terminal.

#### Remarks

*Bivesicula obovata* was described by Shimazu and Machida (1995) on the basis of three specimens, two mature and one immature, from a holocentrid, *S. rubrum*, from Okinawa, Japan. The new specimens, including some from *S. rubrum*, are consistent with their description, most noticeably in the distinctively broad body shape. The original description and figure reports the vitelline follicles as extending just anterior to the arms of the excretory vesicle as opposed to just short of them in the new specimens, but this distinction is considered insignificant.

The specimens of this species were noted as being probably distinct from all others at the time of collection on the basis of their massive body shape. The species has been found by us only at Heron Is. where it has never been common in any of the four hosts in which it has been detected. Only two intact paragenophore specimens and two hologenophores (one front end, one back end) were available to consider the morphology of this species; the single specimens from *E. fasciatus* and *E. undulatostriatus* were consumed entirely for molecular analysis. In terms of *cox*1 sequence data, this species is closest, by far, to *B. claviformis* with which it forms a strongly supported clade. However, Fig. 6B shows the clear distinction in body shape for the two intact GBR specimens together with that from the original description relative to 95 samples of *B. sheni* n.sp., including a single (non-paragenophore) specimen from *S. rubrum* from Heron Is. Surprisingly, despite the ease with which these forms are distinguishable on the basis of body shape, no other morphometric character was found that clearly distinguishes the two species.

**Fig. 8.**
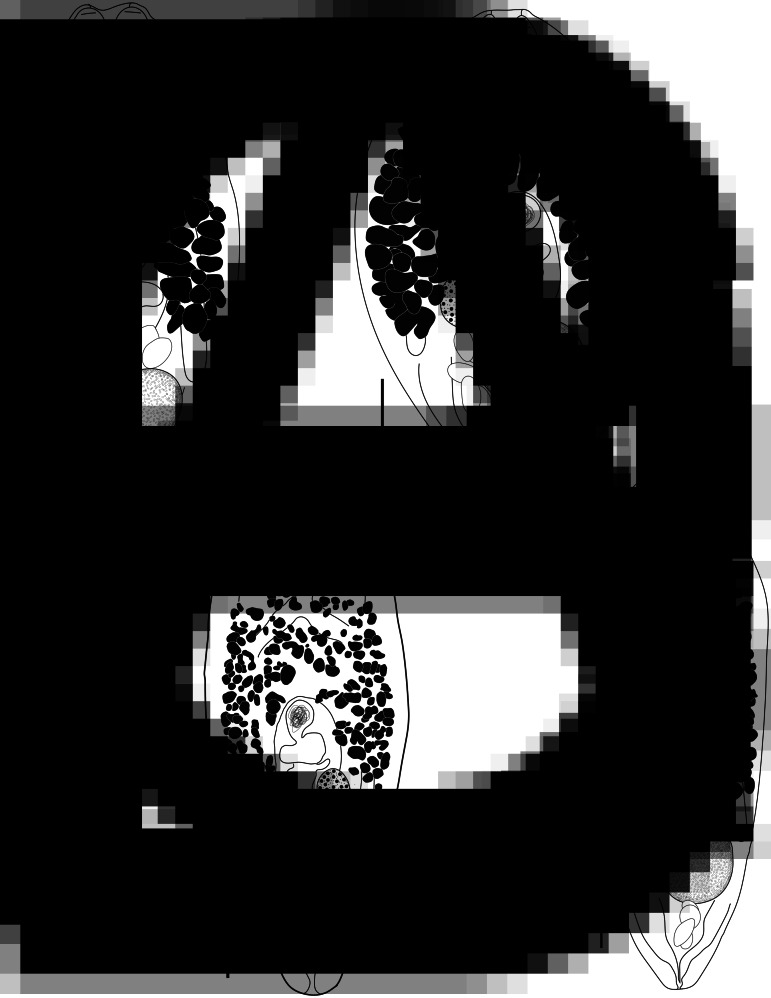
*Bivesicula polynesiensis* n.sp. (A) Holotype from *Sargocentron diadema* from Gambier Archipelago (paragenophore, *cox*1 OTU 3), (B) from *Sargocentron microstoma* from Society Islands (paragenophore, *cox*1 OTU 3), (C) from *Epinephelus fasciatus* from Australs Archipelago (paragenophore, *cox*1 OTU 3), (D) from *Cephalopholis urodeta* from Society Islands (paragenophore, *cox*1 OTU 3). Scale bars = 200 *μ*m.

***Bivesicula polynesiensis*** n.sp. (Fig. 8)

Type host: *Sargocentron diadema* (Lacepède), Crown squirrelfish (Holocentridae).

Type locality: Gambier Archipelago, French Polynesia.

Other hosts: Holocentridae – *Neoniphon sammara* (Forsskål) Sammara squirrelfish; *Sargocentron caudimaculatum* (Rüppell), Silverspot squirrelfish; *Sargocentron microstoma* (Günther), Smallmouth squirrelfish; *Sargocentron spiniferum* (Forsskål), Sabre squirrelfish. Serranidae – *Cephalopholis urodeta* (Forster), Darkfin hind; *Epinephelus fasciatus* (Forsskål), Blacktip grouper.

Other localities: Australs Archipelago, French Polynesia (Tubuai); Society Archipelago, French Polynesia (Moorea).

Site in host: Intestine.

Prevalences: Holocentridae – *N. sammara* 1 of 1 Gambier Archipelago; *S. diadema*, 1 of 1 Gambier Archipelago; *S. spiniferum*, 1 of 2 Gambier Archipelago; *S. microstoma*: 1 of 6 Society Archipelago (Moorea); *S. caudimaculatum*: 1 of 12 Society Archipelago (Moorea). Serranidae – *Epinephelus fasciatus*: 1 of 1 Australs Archipelago (Tubuai); *Cephalopholis urodeta*: 1 of 4 Society Archipelago (Moorea).

Deposited material: MNHN Holotype, HEL1772; 21 paratypes, HEL1773–93; NHMUK four paratypes, 2022.2.15.12–15; QM 14 paratypes, G239667–80.

Representative DNA sequences: Partial *cox*1 mtDNA, 14 sequences (all submitted to GenBank, OM456648–61); ITS2 rDNA, five sequences (all submitted to GenBank, OM523342–46); partial 28S rDNA, three sequences (all submitted to GenBank, OM459992–94).

ZooBank LSID: urn:lsid:zoobank.org:act:81A39CE6-2C43-4744-9BF3-0BFE1BB66C33.

Etymology: The species is named for its type and so far, only known locality, French Polynesia.

*Description*. 44 specimens examined; description based on all material listed above. Measurements given below are for 33 entire, measured specimens, all paragenophores. Measurements for individual host/locality combinations are given in Supplementary Table 3.

Body shape variable, from relatively elongate, with almost parallel sides to far more rounded and distinctly fusiform, 840–1277 × 383–658 (1042 × 482); length/width ratio 1.60–2.97 (2.21). Tegument covered with minute spines throughout. Eyespot pigment dispersed widely at level of oesophagus. Prepharynx short. Pharynx robust, 98–188 × 109–182 (144 × 139), typically approximately as long as wide but highly variable; length/width ratio 0.82–1.36 (1.03). Oesophagus sinuous, 43–156 (84) long, often heavily obscured by vitelline follicles. Caeca blind, of equal length, reaching to 224–372 (308), or 23.2–36.1 (29.6) % body length, from posterior extremity of body. Testis single, entire, post-equatorial, at level of ends of intestinal caeca, 148–240 × 126–219 (185 × 165), 546–942 (687), or 61.4–73.8 (65.9) % body length, from anterior extremity. External seminal vesicle rounded, sometimes extending clearly anterior to cirrus-sac, usually entirely dorsal to cirrus-sac. Cirrus-sac median, orientated antero-posteriorly, filled with prostatic cells, 185–293 × 115–272 (239 × 157), 348–670 (442), or 37.0–52.5 (42.3) % body length, from anterior extremity. Internal seminal vesicle entire. Pars prostatica diverticulate and complex. Genital pore median, close to level of posterior margin of cirrus-sac. Ovary subspherical, unlobed, dorso-dextral to cirrus-sac, 70–125 × 55–100 (97 × 80), 272–459 (372), or 28.7–42.1 (35.6) % body length, from posterior extremity. Uterine seminal receptacle frequently visible. Vitelline follicles variable in size and density, distributed from 127–197 (159), or 12.8–19.6 (15.3) % body length, from anterior extremity, typically almost at level of or exceeding anterior arms of excretory vesicle but also frequently leaving tips clearly exposed, to 280–472 (374), or 30.9–42.3 (35.8) % body length, from posterior end of body, usually terminating distinctly anterior to ends of intestinal caeca, rarely extending to their termination; total vitelline field 370–700 (510) long or 38.8–54.8 (48.9) % of body length. Uterus passes to close to posterior extremity before passing anteriorly to open at common genital pore. Eggs 64–83 × 38–52 (77 × 45). Excretory vesicle V-shaped; arms pass latero-dorsally to testis, terminating close to or distinctly posterior to posterior margin of pharynx, 135–203 (170), or 11.8–21.2 (16.5) % body length, from anterior extremity. Excretory pore terminal.

#### Remarks

As for *B. sheni* n.sp., there is a clear tendency for specimens of this species from holocentrids to be less elongate than those from serranids (Fig. 6C), although there is overlap in body shape for the smaller specimens. If specimens from holocentrids and serranids for *B. sheni* n.sp. and *B. polynesiensis* n.sp. are plotted together, those from holocentrids of the two putative species are more similar to each other in body shape than to those of the same species (Fig. 6D). In addition, and again similar to the findings for *B. sheni* n.sp., 3 of 13 specimens measured from serranids are longer than the longest of 20 specimens from holocentrids suggesting that total size is also affected by host identity.

This species is clearly genetically close to *B. claviformis*, *B. cephalopholicola* n.sp., *B. obovata,* and *B. sheni* n.sp. In the *cox*1 analysis it forms a clade weakly sister to the highly supported clade of *B. sheni* n.sp. + *B. obovata* and *B. claviformis.* The hosts of all four putative species broadly overlap and all have been found in *E. fasciatus*. In terms of morphology, *B. polynesiensis* n.sp. is easily distinguished from *B. obovata* on the basis of the broad body shape of the latter. It is far more similar to *B. claviformis* and *B. sheni* n.sp. Plotting of pharynx length relative to body length suggests a tendency for the pharynx to be larger in *B. polynesiensis* n.sp. than in the other two species; the effect is clearest in larger specimens but there is significant overlap, so this does not create a reliable basis for distinction of the species. Thus, *B. polynesiensis* n.sp. is recognized as a species explicitly cryptic relative to *B. claviformis* and *B. sheni* n.sp. and designated type specimens are all hologenophores and paragenophores.

**Fig. 9.**
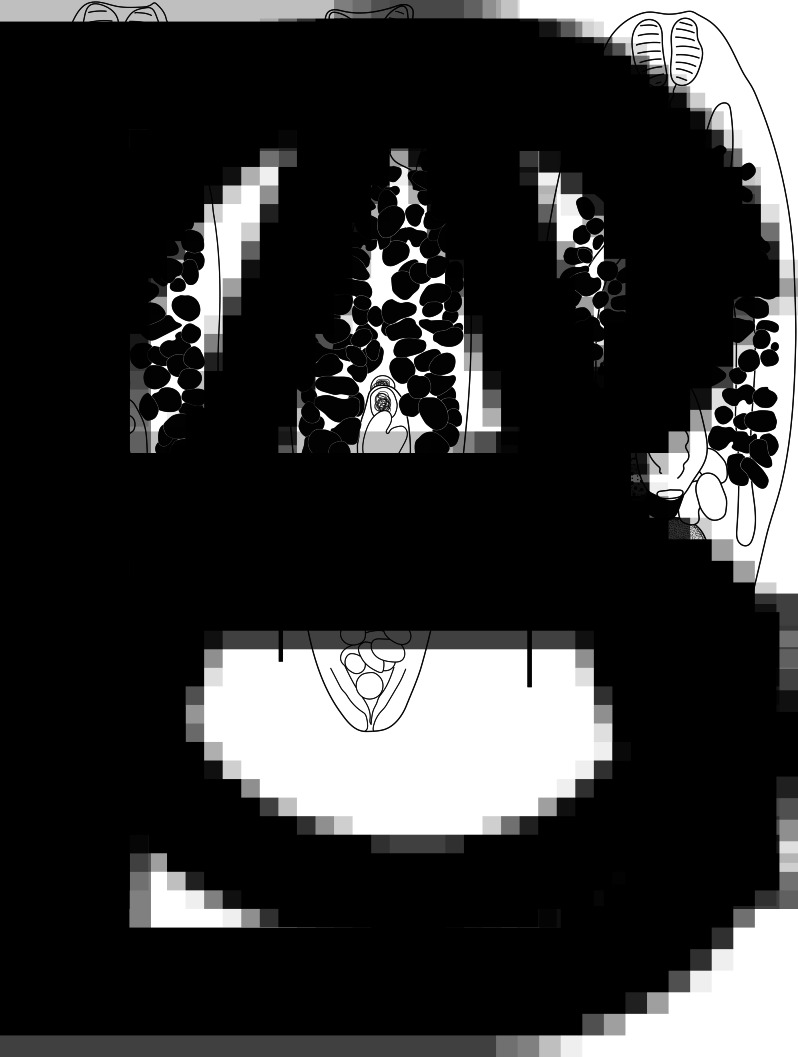
*Bivesicula cephalopholicola* n.sp. and *B. nana* n.sp. (A) Holotype, (B) paratype *B. cephalopholicola* n.sp. from *Cephalopholis boenak* from Lizard Is., (C) Holotype *B. nana* n.sp. from *Epinephelus maculatus* from Lizard Is. Scale bars = 200 *μ*m.

***Bivesicula cephalopholicola*** n.sp. (Fig. 9A and B)

Syn. *B. claviformis* of Justine *et al*. (2010)

Type host: *Cephalopholis boenak* (Bloch), Chocolate hind (Serranidae)

Type locality: Lizard Is., northern GBR.

Other hosts: Serranidae – *Cephalopholis cyanostigma* (Valenciennes), Bluespotted hind. *Cephalopholis microprion* (Bleeker), Freckled hind.

Other localities: New Caledonia.

Site in host: Pyloric caeca and intestine.

Prevalences: *C. boenak* 2 of 12 Lizard Is., 3 of 18, New Caledonia; *C. cyanostigma* 2 of 54 Lizard Is.; *C. microprion* 8 of 9, Lizard Is.

Deposited material: QM Holotype, G239681; 13 paratypes, G239682–94; MNHN four vouchers, HEL1758–61; NHMUK two paratypes, 2022.2.15.1–2.

Representative DNA sequences: Partial *cox*1 mtDNA, four sequences (all submitted to GenBank, OM456615–18); ITS2 rDNA, four sequences (two submitted to GenBank, OM523325–26); partial 28S rDNA, two sequences (both submitted to GenBank, OM459978–79).

ZooBank LSID: urn:lsid:zoobank.org:act:197A691D-D110-48F1-891D-961699662D7C.

Etymology: The species name reflects the only known genus of serranids in which it has been found, *Cephalopholis.* The name should be treated as a noun.

*Description*. 20 specimens examined; measurements are of 17 gravid specimens from Lizard Is. and New Caledonia. Measurements for individual host/locality combinations are given in Supplementary Table 4.

Body relatively elongate, typically with almost straight sides, 883–1562 × 284–550 (1159 × 384); length/width ratio 2.59–3.90 (3.04). Tegument covered with minute spines throughout. Eyespot pigment dispersed widely at level of oesophagus. Prepharynx short. Pharynx robust, usually slightly wider than long, 98–165 × 109–165 (118 × 132); length/width 0.72–1.02 (0.89). Oesophagus sinuous, 41–146 (107) long. Caeca blind, of equal length, reaching to 231–527 (323), or 22.4–33.7 (27.7) % body length, from posterior extremity of body. Testis single, entire, post-equatorial, at level of ends of intestinal caeca, 86–187 × 81–183 (134 × 119), or 62.9–77.2 (69.8) % body length, from anterior extremity. External seminal vesicle rounded, sometimes extending clearly anterior to cirrus-sac, often entirely dorsal to cirrus-sac. Cirrus-sac median, orientated antero-posteriorly, filled with prostatic cells, 135–280 × 75–158 (183 × 101), 437–750 (584), or 46.1–53.8 (50.5) % body length, from anterior extremity. Internal seminal vesicle entire. Pars prostatica diverticulate and complex. Genital pore median, close to level of posterior margin of cirrus-sac. Ovary subspherical, unlobed, dorso-dextral to cirrus-sac, 65–166 × 52–121 (90 × 78), 274–624 (378), or 27.4–39.9 (32.3) % body length, from posterior extremity. Uterine seminal receptacle frequently visible. Vitelline follicles consistently robust, distributed from 158 to 253 (197), or 13.2–21.9 (17.3) % body length, from anterior extremity, close to level of anterior extent of arms of excretory vesicle but always leaving tips clearly exposed, to 263–471 (349), or 25.6–35.7 (30.0) % body length, from posterior end of body, usually terminating only slightly anterior to ends of intestinal caeca and sometimes extending to their termination; total vitelline field 425–885 (614) long or 48.1–57.0 (52.7) % of body length. Uterus passes to close to posterior extremity before passing anteriorly to open at common genital pore. Eggs 74–92 × 36–50 (81 × 44). Excretory vesicle V-shaped; arms pass latero-dorsally to testis, terminating distinctly posterior to posterior margin of pharynx, 122–195 (148), or 10.4–15.1 (12.9) % body length, from anterior extremity. Excretory pore terminal.

#### Remarks

This species has been detected in three species of *Cephalopholis* at Lizard Is. on the northern GBR and in one of the same species from New Caledonia. The New Caledonian identification is not supported by molecular data, but the shared host and comparable morphology lead us to identify them as the same species with confidence. Despite the examination of 64 individuals of two susceptible *Cephalopholis* species at Heron Is., this species has so far not been detected there, although the most commonly infected host species, *C. microprion*, has not been examined there. Single infections of species of *Bivesicula* in species of *Cephalopholis* have been detected in French Polynesia and at Okinawa, but, on the basis of sequence data, neither relates to this species.

The morphology of this form is clearly broadly comparable to that of *B. claviformis*, *B. obovata, B.sheni* n.sp. and *B. polynesiensis* n.sp. Relative to these species it is moderately morphologically, biologically and genetically distinctive. *cox*1 sequences of *B. cephalopholicola* n.sp. differ from all other species at a minimum of 45 base positions. Within Clade 1, based on *cox*1 and 28S rDNA, it is sister to all other species except for the form from Bali. Morphologically it is far narrower than *B. obovata*. It is similar in shape to *B. claviformis, B.sheni* n.sp. and *B. polynesiensis* n.sp. but has a generally proportionally smaller cirrus-sac (although there is considerable overlap in measurements) (Fig. 6E). The data for the GBR suggest that the species is restricted to the serranid genus *Cephalopholis* and, that within the known range, no other bivesiculids infect the same fishes. These conclusions are supported by the findings from New Caledonian serranids. Specimens identified as *B. cephalopholicola* n.sp. were found only in *C. boenak*; absence of infections of morphologically comparable specimens in large numbers of multiple species of *Epinephelus* supports the inferred restriction of *B. cephalopholicola* n.sp. to species of *Cephalopholis*.

***Bivesicula nana*** n.sp. (Fig. 9C)

Type host *Epinephelus maculatus* (Bloch) Serranidae.

Type locality: Lizard Is., northern GBR.

Site in host: Pyloric caeca and intestine.

Prevalence: 1 of 10 *E. maculatus.*

Deposited material: QM Holotype, G239695; seven paratypes, G239696–702.

ZooBank LSID: urn:lsid:zoobank.org:act:E2AE6C04-189A-4442-B4C1-FA1AB5AA1B6B.

Etymology: The name reflects the diagnostically tiny size of this species. The name should be treated as a noun.

*Description*. Eight specimens examined; measurements are of 5 gravid specimens.

Body minute, fusiform, 502–647 × 175–246 (575 × 211); length/width ratio 2.48–2.88 (2.74). Tegumental spines not detected. Eyespot pigment dispersed widely at level of oesophagus. Prepharynx short. Pharynx robust, always slightly wider than long, 46–62 × 55–65 (53 × 59); length/width 0.82–0.95 (0.89). Oesophagus sinuous, 72–79 (75) long. Caeca blind, of equal length, reaching to 139–162 (153), or 24.7–29.1 (26.6) % body length, from posterior extremity of body. Testis single, entire, post-equatorial, at level of ends of intestinal caeca, 54–77 × 44–69 (66 × 58), 366–459 (411), or 70.2–72.9 (71.6) % body length, from anterior extremity. External seminal vesicle rounded, sometimes extending clearly anterior to cirrus-sac, sometimes entirely dorsal to cirrus-sac. Cirrus-sac median, orientated antero-posteriorly, filled with prostatic cells, 92–115 × 55–68 (105 × 61), 252–327 (286), or 47.6–50.5 (49.7) % body length, from anterior extremity. Internal seminal vesicle entire. Pars prostatica diverticulate and complex. Genital pore median, close to level of posterior margin of cirrus-sac. Ovary subspherical, unlobed, dorso-dextral to cirrus-sac, 28–38 × 26–37 (33 × 29), 155–212 (185), or 30.9–33.6 (32.1) % body length, from posterior extremity. Uterine seminal receptacle frequently visible. Vitelline follicles robust, distributed from 85 to 115 (102), or 16.4–20.0 (17.7) % body length, from anterior extremity, close to level of anterior extent of arms of excretory vesicle but always leaving tips clearly exposed, to 155–213 (181), or 30.0–32.9 (31.4) % body length, from posterior end of body, terminating distinctly anterior to ends of intestinal caeca; total vitelline field 259–323 (293) long or 47.6–52.9 (50.9) % of body length. Uterus passes to close to posterior extremity before passing anteriorly to open at common genital pore. Eggs 56–59 × 32–35 (57 × 33). Excretory vesicle V-shaped; arms pass latero-dorsally to testis, terminating distinctly posterior to posterior margin of pharynx, 73–83 (77), or 12.7–15.3 (13.5) % body length, from anterior extremity. Excretory pore terminal.

#### Remarks

This is the only species dealt with here for which molecular data are lacking. The species was found only once, in *E. maculatus* at Lizard Is. However, the morphology of this form is so distinctive that a new species can be proposed for it with confidence. The species generally resembles *B. claviformis* and its relatives and, like them, infects a serranid. The distinctiveness of the species relates almost entirely to its size. The five good quality gravid specimens range in length only from 502 to 647 *μ*m. This small size is not unprecedented in the family. *Bivesicula neglecta* is reported at only 437–675 *μ*m, *B. unexpecta* at only 506–633 *μ*m and *B. caribbensis* Cable & Nahhas, 1962 at 654–1020 *μ*m. However, these three species are all immediately distinct from the present form in having bodies that are nearly round (length/width ratio of <1.50 in contrast to that of 2.48–2.88 for *B. nana* n.sp.). None of the species with body shapes and proportions generally comparable to the present form have ever been reported in the size range reported here. Confidence in the value of this morphological distinction is strengthened by comparison with 119 specimens interpreted as representing *B. sheni* n.sp. Of these, 17 were immature but at least as large as the smallest gravid *B. nana* n.sp. and 14 were larger than all of the *B. nana* n.sp. The 90 gravid *B. sheni* n.sp. all exceeded 800 *μ*m. These distinctions, illustrated in Fig. 6F, included specimens of both *B. sheni* n.sp. and *B. nana* n.sp. from the same individual *E. maculatus* so that there is no basis for host-induced morphological distinction in these differences. In addition to the difference in body size, the eggs of *B. nana* n.sp. are also noticeably smaller than those of any of the other species considered here. Although the eggs of *B. australis*, *B. megalopis* Shen, 1982 and *B. tarponis* Sogandares-Bernal & Hutton, 1959 are all reported at about the size of the eggs of *B. nana* n.sp., none has as small a maximum length (just 59 *μ*m). Again, none of these species shows any general resemblance to *B. nana* n.sp.

***Bivesicula* Clade 2**

**Fig. 10.**
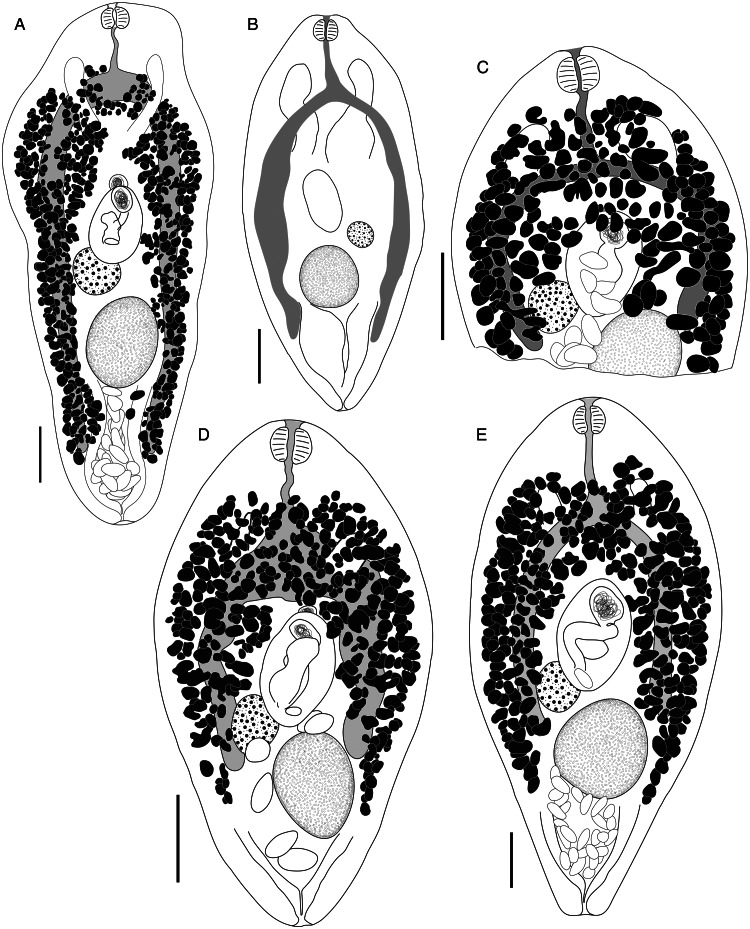
*Bivesicula palauensis* Shimazu and Machida, 1995. (A) from *Variola albimarginata*, Okinawa (paragenophore, *cox*1 OTU 11), (B) from *Epinephelus morrhua,* New Caledonia. *Bivesicula gymnothoracis* Shimazu and Machida, 1995, (C) from *E. fasciatus*, Okinawa, Japan (paragenophore, *cox*1 OTU 12), (D) from *Epinephelus fasciatus*, Minabe, Japan (paragenophore, *cox*1 OTU 13), E.*Bivesicula novaecaledoniensis* n.sp., Holotype from *Epinephelus chlorostigma*, New Caledonia (paragenophore, *cox*1 OTU 10). Scale bars = 200 *μ*m.

***Bivesicula palauensis*** Shimazu and Machida, 1995 (Fig. 10A andB)

Type host: *Variola louti* (Forsskål), Yellow-edged lyretail (Serranidae).

Other hosts: *Epinephelus polyphekadion* (Bleeker) (reported as *E. microdon*), Camouflage grouper (Serranidae).

Type locality: Ngeremlengui, Palau.

Other localities: Koror, Palau.

#### This study

Hosts: Serranidae – *Epinephelus areolatus* (Forsskål), Areolate grouper; *Epinephelus morrhua* (Valenciennes), Comet grouper; *Variola albimarginata* Baissac, White-edged lyretail.

Localities: Okinawa, Japan (Tomari Fish Market); New Caledonia.

Site in host: Intestine.

Prevalences: *E. areolatus* 1 of 1 Okinawa; *E. morrhua* 1 of 4, New Caledonia; *V. albimarginata* 1 of 2, Okinawa.

Deposited material: MPM four vouchers, 21846–2; MNHN three vouchers, HEL1769–71.

Representative DNA sequences: Partial *cox*1 mtDNA, four sequences (all submitted to GenBank, OM456644–47); ITS2 rDNA, two sequences (both submitted to GenBank, OM523340–41); partial 28S rDNA, two sequences (both submitted to GenBank, OM459990–91).

ZooBank LSID: urn:lsid:zoobank.org:act:95B63AF2-A176-4E25-AE5B-DA94A2D0E5C0.

Measurements. See Supplementary Table 5.

#### Remarks

This species was described effectively by Shimazu and Machida (1995) as a distinctive species. As such, no new description is provided, but new measurements and a figure for a new host/locality combination are provided. *Bivesicula palauensis* is among the largest species of *Bivesicula,* reported to over 3 mm, and also distinctive in its small pharynx relative to overall body size and especially in having the vitelline follicles extend to <15% of the body length from the posterior extremity. Most species in the genus have the vitelline follicles at least 20% of the body length from the posterior extremity. The only other species comparable in this character is *B. neglecta*, a much smaller and highly squat species that is otherwise not confusable with *B. palauensis*. Of six specimens of *B. palauensis* collected from Okinawa, two were sequenced as hologenophores, two damaged specimens were consumed entirely for sequencing, and two moderately well-fixed specimens were stained and mounted intact. From New Caledonia a single gravid specimen and two immatures from one individual *E. morrhua* were reported by Justine *et al*. (2010) as an unidentified species of *Bivesicula*. All these specimens are consistent with the original description of the species, especially in the close approach of the vitelline follicles to the posterior extremity.

New specimens were found in species of *Variola* and *Epinephelus*, just as originally reported by Shimazu and Machida (1995), although from different species. Although the specimens are smaller than reported by Shimazu and Machida (1995), they are still larger than almost all the other specimens of other species of *Bivesicula* reported here. The new samples are confidently identified as *B. palauensis*, extending the known distribution from Palau to Okinawa and New Caledonia. Sequence data suggest that this species is unambiguously different from all the other species of *Bivesicula* for which comparable data are now available.

***Bivesicula gymnothoracis*** Shimazu and Machida, 1995 (Fig. 10C and D)

Type host: *Gymnothorax kidako* (Temminck & Schlegel), Kidako moray (Muraenidae).

Type locality: Fukaura, Ehime Prefecture, Japan.

#### This study

Hosts: Muraenidae – *G. kidako*; Serranidae – *Epinephelus fasciatus* (Forsskål), Blacktip grouper.

Localities: Minabe, Japan (Minabe fish market); Okinawa, Japan (Tomari Fish Market).

Site in host: Intestine.

Prevalences: Minabe *G. kidako* 2 of 4; *E. fasciatus* 3 of 3; Tomari *E. fasciatus* 3 of 5.

Deposited material: MPM 27 vouchers, 21843–5; NHMUK five vouchers, 2022.2.15.3–7; QM 12 vouchers, G239703–14.

Representative DNA sequences: Partial *cox*1 mtDNA, nine sequences (six submitted to GenBank, OM456629–34); ITS2 rDNA, four sequences (three submitted to GenBank, OM523333–35); partial 28S rDNA, two sequences (both submitted to GenBank, OM459984–85).

ZooBank LSID: urn:lsid:zoobank.org:act:0E83AF93-7C97-4777-B0B5-4CEDE9282360.

Measurements. See Supplementary Table 6.

#### Remarks

This species was described effectively by Shimazu and Machida (1995) on the basis of three specimens taken from *G. kidako* from Fukaura, Ehime Prefecture, Japan. New specimens were obtained from *G. kidako* from the Minabe fish market, a little under 300 km to the north-east of Fukaura. Although the original specimens are all larger than those reported from *G. kidako* here (3.08–4.56 mm long v.a max. of 2.99 mm), the morphological agreement and the identification is clear. Specimens of this species were also collected from the serranid *E. fasciatus* (Fig. 10C and D) at both Minabe and in Okinawa. This identification came as a great surprise, given the host distinction, and was revealed initially only by the generation of identical *cox*1 sequences for specimens from the two fish species. The size range of the samples from the two host species is almost non-overlapping. Nine of 10 good specimens from *G. kidako* are 2.42–2.99 mm long whereas 15 from *E. fasciatus* are 1.02–1.36 mm long. However, a single smaller specimen, 1.29 mm long detected from *G. kidako*, agrees well with those from *E. fasciatus*. Although morphologically comparable, *B. palauensis* and *B. gymnothoracis* are immediately distinguishable by the more posteriorly extensive vitellarium of the former species.

***Bivesicula novaecaledoniensis*** n.sp. (Fig. 10E)

Type host: *Epinephelus chlorostigma* (Valenciennes), Brownspotted grouper.

Other hosts: *Epinephelus fasciatus* (Forsskål), Blacktip grouper.

Type locality: New Caledonia.

Prevalence: *E. chlorostigma* 3 of 3*; E.fasciatus* 3 of 61.

Deposited material: MNHN Holotype, HEL1762; 22 paratypes, HEL1762–8; NHMUK two paratypes, 2022.2.15.8–9; QM 11 paratypes, G239715–25.

Representative DNA sequences: Partial *cox*1 mtDNA, four sequences (all submitted to GenBank, OM456636–39); ITS2 rDNA, two sequences (one submitted to GenBank, OM523337); partial 28S rDNA, two sequences (one submitted to GenBank, OM459987).

ZooBank LSID: urn:lsid:zoobank.org:act:B7C1FD3C-150F-423C-9865-EBF2DD604740.

Etymology: The name is derived from the type and only known locality for this species, New Caledonia.

*Description*. 38 specimens examined; measurements below are of 22 gravid specimens combined from both New Caledonian hosts. Measurements for individual host/locality combinations given in Supplementary Table 7.

Body distinctly pyriform, 997–2046 × 521–1002 (1561 × 758); length/width ratio 1.56–2.49 (2.08). Tegument covered with minute spines throughout. Eyespot pigment dispersed widely at level of oesophagus. Prepharynx short. Pharynx small, always at least slightly wider than long, 67–116 × 87–129 (90 × 111); length/width 0.71–0.96 (0.81). Oesophagus sinuous, 116–252 (195) long. Caeca blind, of equal length, reaching to 311–844 (500), or 17.7–41.4 (32.1) % body length, from posterior extremity of body. Testis single, entire, post-equatorial, at level of ends of intestinal caeca, 166–374 × 150–343 (272 × 247), 602–1167 (927), or 55.5–64.6 (59.6) % body length, from anterior extremity. External seminal vesicle rounded, sometimes extending clearly anterior to cirrus-sac, often entirely dorsal to cirrus-sac. Cirrus-sac median, orientated antero-posteriorly, filled with prostatic cells, 210–414 × 141–292 (324 × 220), 380–735 (565), or 28.6–42.6 (36.3) % body length, from anterior extremity. Internal seminal vesicle entire. Pars prostatica diverticulate and complex. Genital pore median, close to level of posterior margin of cirrus-sac. Ovary subspherical, unlobed, dorso-dextral to cirrus-sac, 86–188 × 74–170 (137 × 114), 395–921 (655), or 37.1–45.1 (41.8) % body length, from posterior extremity. Uterine seminal receptacle frequently visible. Vitelline follicles consistently robust, distributed from 106 to 294 (195), or 10.0–15.2 (12.5) % body length, from anterior extremity, almost always concealing anterior tips of excretory vesicle arms, to 297–603 (405), or 19.2–32.8 (25.9) % body length, from posterior end of body, always terminating well posterior to ends of intestinal caeca; total vitelline field 564–1241 (961) long or 56.6–68.8 (61.6) % of body length. Uterus passes close to posterior extremity before passing anteriorly to open at common genital pore. Eggs 74–95 × 38–50 (87 × 45). Excretory vesicle V-shaped; arms pass latero-dorsally to testis, terminating distinctly posterior to posterior margin of pharynx, 151–333 (242), or 11.3–18.9 (15.5) % body length, from anterior extremity. Excretory pore terminal.

#### Remarks

This species falls clearly in Clade 2, with *B. palauensis* and *B. gymnothoracis*, in all molecular analyses. It is known from *E. chlorostigma* from New Caledonia on the basis of 35 specimens and multiple sequences and just three specimens from *E. fasciatus*, not sequenced, from the same locality. Despite the limited samples from *E. fasciatus*, their morphology is strongly consistent with that of the specimens from *E. chlorostigma*.

This species is easily distinguishable from *B. palauensis* on the basis of its significantly less posteriorly extensive vitelline follicle distribution. However, it is strikingly similar to *B. gymnothoracis* which is surprising given the substantial molecular distinctions between the two (81–84 *cox*1, 7 ITS2 rDNA and 11 28S rDNA base positions differences between the two species), equivalent to the differences between multiple combinations of other clearly morphologically distinct species. Just one character showed some distinction between the two – the anterior extent of the vitelline follicles relative to the pharynx. Although measurements for this character overlap extensively for the smallest specimens, they diverge substantially for larger specimens. No basis for distinction was found in body shape, the posterior extent of the vitelline follicles, size of the pharynx, or egg size. Significantly, the two species have an overlapping host range (both infect *E. fasciatus*). In the light of the criteria for the recognition of species, the failure of *B. gymnothoracis* and *B. novaecaledoniensis* n.sp. to form an exclusive clade (*B. novaecaledoniensis* n.sp. is consistently identified as sister to *B. palauensis*) means that a new species is required for these specimens. Given that they are morphologically cryptic, the designated type specimens are all paragenophores.

Justine *et al*. (2010) examined 61 individual *E. fasciatus* from New Caledonian waters for internal parasites and, for bivesiculids, found only the three specimens here identified as *B. novaecaledoniensis* n.sp. in three individual fishes. They also examined 38 *E. maculatus* and 18 *E. merra* which harbour *B. sheni* n.sp. on the GBR. These data suggest the possible absence of *B. sheni* n.sp., *B. claviformis* and *B. obovata* in New Caledonia, but their replacement with *B. novaecaledoniensis* n.sp. which has not been found on the GBR. This possible absence is surprising given *B. claviformis* has been reported from Fiji by Manter (1961).

## Discussion

### Species recognition

If a simple morphological species concept was employed, it would be reasonable to recognize six species of *Bivesicula* in this collection from holocentrids, muraenids and serranids: *B. cephalopholicola* n.sp., *B. claviformis, B.gymnothoracis*, *B. nana* n.sp., *B. obovata* and *B. palauensis*. This classification would, however, have had difficulty recognizing host-induced morphological variation for some of the species. Molecular data enable the recognition of three additional largely cryptic species, *B. novaecaledoniensis* n.sp., *B. polynesiensis* n.sp. and *B. sheni* n.sp., suggest the presence of a fourth on the basis of a sequence of a worm from Bali, confirm the distinction of all the others except for *B. nana* n.sp. which remains unsequenced, and allow the recognition of host-induced morphological variation.

The final taxonomic hypothesis is heavily dependent on the interpretation of *cox*1 sequence data. Most importantly, combinations of samples ultimately interpreted as *B. claviformis*, *B. sheni* n.sp. and *B. obovata* differ at 44–47, 38–43 and 21–24 base positions in the *cox*1 alignment. However, they have partly identical ITS2 rDNA sequences that never differ at more than a single base position and for 28S rDNA sequences the same three combinations differ at only 1–2 base positions. On the basis of evidence relating to two previously described species it appears that ITS2 and 28S rDNA are poor markers for closely related species of *Bivesicula*. *Bivesicula neglecta* and *B. unexpecta* are clearly morphologically distinct and infect fishes of separate families (Pomacentridae and Apogonidae) in sympatry. They differ at just one base position in the ITS2 rDNA alignment (Trieu *et al*., 2015) and three base positions of 28S rDNA (this study) but new data here shows that they differ at 35 base positions in the *cox*1 alignment. The case of the delineation of *B. sheni* n.sp. and *B. obovata* in this study is similarly informative. The two are readily morphologically distinguishable, although in this case they infect an overlapping range of fishes in sympatry. The two forms have identical ITS2 rDNA sequences and differ by just one base position in 28S rDNA data but are clearly distinguished at 21–24 base positions in the *cox*1 dataset. Despite its extensive successful use to delineate species of many trematode families (see Blasco-Costa *et al*., 2016), it appears that ITS2 and 28S rDNA sequences are frequently inadequate markers for species delineation in this family. A comparable problem was identified recently for certain Aporocotylidae (Cutmore *et al*., 2021).

This study applied the criteria proposed by Bray *et al*. (2022) for recognition of species (reciprocal monophyly plus distinction in either morphology or host-specificity). Testing for reciprocal monophyly using only *cox*1 mtDNA sequences has the problem that this marker does not recombine easily and is highly prone to rapid divergence so that what might best be ultimately interpreted as regional populations may be strongly reciprocally monophyletic. It is for that reason that, for an effective basis for species recognition, Bray *et al*. (2022) called for the additional fulfilment of at least one of the two additional criteria. By this approach they recognized multiple monophyletic, regionally distributed populations as relating to a single lepocreadiid species (*Preptetos laguncula* Bray & Cribb, 1996) because, ultimately, they formed a single clade, were morphologically indistinguishable, and infected exactly the same host species. In the present system the two most problematic combinations of species are resolved on the following bases: *B. sheni* n.sp. and *B. obovata* form a single clade, infect an overlapping range of fishes but are also reciprocally monophyletic and morphologically distinguishable; *B. claviformis*, *B. sheni* n.sp. and *B. polynesiensis* n.sp. are morphologically indistinguishable (*B. polynesiensis* n.sp. is weakly distinguishable) and infect the same hosts, but each is reciprocally monophyletic with respect to each other and no combination of the three forms a monophyletic clade. Critical to this topological issue is the presence of the morphologically distinguishable *B. obovata* forming a clade with *B. sheni* n.sp.; without the presence of this species there would be a case to recognize *B. claviformis*, *B. sheni* n.sp. and *B. polynesiensis* n.sp. as a single widespread species. Were *B. claviformis* and *B. sheni* n.sp. to be considered a single species then the problem of cryptic species in the same sympatric hosts (on the GBR) would be removed. However, another discrepancy would arise in that *B. claviformis* as recognized here is a widespread species with a commensurate population structure – distinct *cox*1 populations in Japan, Ningaloo and the GBR distinguished at a level (17–23 bases positions) readily interpreted as intraspecific variation. The presence of one of these populations on the GBR together with a second form (here recognized as *B. sheni* n.sp.) that differs at 44–46 base positions in the *cox*1 dataset seems inconsistent with the radiation of a single species. Notably, Bray *et al*. (2022) reported that two species of *Preptetos* are each represented by two distinct *cox*1 populations on the northern GBR. The final distinction between the recognition of those samples of *Preptetos* as representing single species and the specific distinction between *B. claviformis* and *B. sheni* n.sp. recognized here is dependent on the finding that the *Preptetos* lineages ultimately formed monophyletic assemblages whereas *B. claviformis* and *B. sheni* n.sp. do not.

The subtleties of species recognition discussed here led to the conclusion that species of *Bivesicula* are so morphologically conservative that their morphology often reflects species level differences only weakly, if at all. This point is emphasized by the finding that species of *Bivesicula* Clade 3 form a clade with the highly distinctive genus *Paucivitellosus* rather than with the other clades of *Bivesicula.* This topology suggests that *Bivesicula* will ultimately need division but that the morphological distinctions between the resulting genera will be subtle. Two general explanations suggest themselves for the apparent morphological conservatism of species of *Bivesicula.* The first combines the possibilities that species of *Bivesicula* are unusually morphologically constrained and unvarying for trematodes, that this constraint is exacerbated by the absence of key digenean characters (the suckers) typically used heavily in taxonomy, and the possibility that the taxa concerned are a recently and rapidly diverging group of species. The alternative possibility is that there is little special about the group (apart from the lack of suckers) but that instead it has been studied over the range at a depth which is not yet common for trematodes of fishes of the Indo-Pacific. It is clear that the complexities of systems such as these make the issue of species recognition difficult. It is to be hoped that the stable criterion-based and system-specific approach to recognition of species used here at least has the capacity to make taxonomic proposals consistent.

### Host specificity

The evolving understanding of the host specificity of bivesiculids is proving exceptionally interesting. The dominant paradigm of fish trematode specificity, at least for those of tropical marine fishes, is of oio- or stenoxenicity (Miller *et al*., 2011), the infection of single or closely related species. In this context it was astonishing to find clear evidence of euryxenicity (infection of phylogenetically unrelated species) for four species shared between holocentrids and serranids and one shared by a serranid and a muraenid; in each case the two families belong to different orders of fishes. The five other species in the study have been found only in serranids, but in each case sampling is probably inadequate to allow confidence that the species is restricted to just one family of fishes. The basis of this exceptional pattern of host-specificity is far from clear but must be partly based on the mode of transmission. Cribb *et al*. (1998) provided evidence that transmission of *B. claviformis* on the GBR (probably actually the new species *B. sheni* n.sp.) is *via* small fish which had ingested cercariae and in which the juvenile parasite awaited transmission in the gut. Such a transmission pattern, dependent on ingestion of small fish, is broadly consistent with the diets of a wide range of holocentrids, muraenids and serranids. However, there is no clear explanation for the general absence of infections in the many other families of piscivorous fishes. For example, on the GBR *B. sheni* n.sp. infects two orders of fishes, seven species of *Epinephelus* (Serranidae) and two species of *Sargocentron* (Holocentridae). It has not been found in other well-sampled serranids such as species of *Plectropomus* Oken, which are voracious piscivores as shown by their rich fauna of bucephalids (Bott *et al*., 2013) or in species of *Cephalopholis* which harbour a different species of *Bivesicula* sympatrically (this study). These discrepancies have no clear explanation. The anomalous sharing of unrelated host families might be considered a species-specific character and thus argue against the interpretation that four species share infection of holocentrids and serranids. However, there is no reason *per se* to argue against a modest radiation of a lineage of *Bivesicula* with this exceptional host specificity. The finding that *B. gymnothoracis*, belonging to a clearly distinct lineage of *Bivesicula* species, infects both muraenids and serranids (again fishes of separate orders), supports that idea. The only comparably disjunct host distribution of GBR fish trematodes is that of *Transversotrema borboleta* Hunter and Cribb, 2012, infecting both chaetodontid and lutjanid fishes which are distantly related, but none of many other seemingly suitable hosts (Hunter and Cribb, 2012).

The second aspect of the host-specificity of the group of *Bivesicula* species considered here is the remarkable role of *Epinephelus fasciatus*; seven of the 10 species distinguished here have been found in this fish. No aspect of the biology of *E. fasciatus* suggests why it should be especially susceptible to infection by bivesiculids. Strikingly, *E. fasciatus* is infected by two lineages of species (Clade 1 – *B. claviformis, B.sheni* n.sp., *B. obovata, B.polynesiensis* n.sp. and the form known only from Bali; Clade 2 – *B. gymnothoracis* and *B. novaecaledoniensis* n.sp.). An issue not yet much considered in the structuring of the trematode fauna of Indo-Pacific fishes is that of how widespread fish species are exploited – is it by the same or different species at different localities? There is a developing body of molecular evidence supporting widespread distributions for trematode species (e.g. Aiken *et al*., 2007; Bray *et al*., 2022; Huston *et al*., 2021), although increasingly populations may be divided geographically (as here for *B. claviformis*) (Bray *et al*., 2018, 2022; Cutmore *et al*., 2021). In the present study there is evidence for another pattern, speciation independent of that of the definitive hosts. This system is rendered complex by the conclusion that at least one species (*B. claviformis*) is widespread and shows intraspecific geographic structuring. Certainly, *E. fasciatus* is an especially interesting subject for further analysis over its range. In this context it is noteworthy that Kuriiwa *et al*. (2014) demonstrated that *E. fasciatus* itself has a complex structure of three cryptic lineages that may co-occur in Japanese waters. The relationship that this structure bears to the nature of their trematodes remains to be explored.

### Host-induced phenotypic plasticity

The final aspect of this system that is exceptional and problematic, is the evidence that the morphology of some species of *Bivesicula* varies noticeably with the host in which they develop. The broad experience of trematodes of the gastrointestinal tract of tropical marine fishes, is that such phenotypic plasticity is not common; however, infection of such unrelated hosts is also not common. Perusal of the literature also suggests that the phenomenon is not reported especially frequently. There is a potential interpretative trap in this area; in the absence of molecular or experimental data, phenotypic plasticity might well go unrecognized as such and be interpreted as inter-specific variation. Regardless, there are reports relating to the phenomenon for at least 10 trematode families: Diplostomidae (Pérez-Ponce de León, 1995); Echinostomatidae (Hildebrand *et al*., 2015); Gorgoderidae (Bakke, 1988; Cutmore *et al*., 2010); Haematoloechidae (Kennedy, 1980); Haploporidae (Tonatiuh Gonzalez-Garcia *et al*., 2021); Heterophyidae (Elshazly *et al*., 2007; Fraija-Fernandez *et al*., 2015; Presswell and Bennett, 2020); Microphallidae (Martorelli and Ivanov, 1996); Plagiorchiidae (Blankespoor, 1974); Schistosomatidae (Machadosilva *et al*., 1994); Zoogonidae (Mouahid *et al*., 2012).

The host-induced morphological variation that is seen here has two significant facets. First, the variation occurs in the context of morphology of species of a genus in which distinguishing characters are relatively few; for a start, dimensions and relativities of oral and ventral suckers are not available for analysis. In addition to the host-induced morphological variation, there was considerable intraspecific variation not obviously associated with any biological or geographical variable. Thus, there are multiple sources of intraspecific morphological variation among these taxa, increasing the difficulty of finding reliable characters for interspecific distinction. Second, the remarkable disjunct host distributions of many of these species can easily create a false expectation of the likely presence of separate species which is reinforced by subtle distinctions in morphology. The combination of these effects, together with the complexities of the distribution of the species involved, mean that molecular data are essential for progress with this field.

## Data Availability

The data that support the findings of this study are available from the corresponding author upon reasonable request.

## Supplementary material

For supplementary material accompanying this paper visit https://doi.org/10.1017/S0031182022000282. click here to view supplementary material
