# Supplementary material for: A world of taxonomic pain: cryptic species, inexplicable host-specificity, and host-induced morphological variation among species of Bivesicula Yamaguti, 1934 (Trematoda: Bivesiculidae) from Indo-Pacific Holocentridae, Muraenidae and Serranidae
Source: Parasitology. 2022 Mar 10;149(6):831–53. doi: 10.1017/S0031182022000282 (PMC10090613; doi:10.1017/S0031182022000282)
Supplement: Supplementary file 1 [file S0031182022000282sup001.zip › S0031182022000282sup001.docx]

**Supplementary Table 1**. *Bivesicula claviformis* Yamaguti, 1934 measurements.

| Host family | Serranidae | | | Holocentridae | | | Serranidae | Serranidae | | | Serranidae |
| --- | --- | --- | --- | --- | --- | --- | --- | --- | --- | --- | --- |
| Host species | *E. merra* | | | *S. spiniferum* | | | *E. fasciatus* | *E. fasciatus* | | | *E. tauvina* |
| Locality | Lizard Island | | | Lizard Island | | | Okinawa | Ningaloo | | | Ningaloo |
| n | 4 | | | 4 | | | 1 | 13 | | | 1 |
|  | **Min** | **Max** | **Mean** | **Min** | **Max** | **Mean** |  | **Min** | **Max** | **Mean** |  |
| Body L | 1205 | 1539 | 1358 | 739 | 895 | 843 | 1024 | 938 | 1468 | 1194 | 931 |
| Body W | 413 | 567 | 495 | 383 | 425 | 398 | 563 | 312 | 526 | 431 | 387 |
| Body L / Body W | 2.44 | 2.99 | 2.77 | 1.92 | 2.34 | 2.12 | 1.82 | 2.33 | 3.46 | 2.80 | 2 |
| Pharynx L | 101 | 141 | 118 | 72 | 90 | 85 | 110 | 101 | 161 | 133 | 104 |
| Pharynx W | 116 | 157 | 139 | 90 | 111 | 105 | 146 | 105 | 137 | 127 | 114 |
| Pharynx L / Pharynx W | 0.81 | 0.90 | 0.85 | 0.79 | 0.82 | 0.81 | 0.75 | 0.87 | 1.36 | 1.06 | 1 |
| Oesophagus | 119 | 163 | 148 | 102 | 130 | 120 | 77 | 73 | 123 | 105 | 116 |
| Caeca to posterior end | 278 | 418 | 365 | 191 | 247 | 219 | 306 | 250 | 450 | 328 | 267 |
| Caeca to posterior end as % BL | 21.1 | 30.5 | 26.9 | 23.5 | 27.6 | 25.9 | 29.9 | 23.3 | 31.8 | 27.4 | 28.7 |
| Testis L | 155 | 178 | 165 | 90 | 118 | 107 | 207 | 101 | 220 | 161 | 101 |
| Testis W | 141 | 170 | 154 | 84 | 111 | 99 | 200 | 87 | 204 | 149 | 90 |
| Testis to anterior end | 836 | 1067 | 935 | 518 | 630 | 591 | 656 | 651 | 934 | 783 | 660 |
| Testis to anterior end as % BL | 65.4 | 71.5 | 68.9 | 68.5 | 73.2 | 70.1 | 64.1 | 62.5 | 69.4 | 66.5 | 70.9 |
| Cirrus-sac to anterior end | 520 | 770 | 654 | 347 | 440 | 402 | 444 | 412 | 592 | 512 | 420 |
| Cirrus-sac to anterior end as % BL | 43.2 | 52.2 | 48.0 | 46.1 | 51.1 | 47.7 | 43.4 | 40.3 | 46.1 | 43.0 | 45.1 |
| Cirrus-sac L | 219 | 250 | 234 | 138 | 164 | 157 |  | 203 | 309 | 258 | 196 |
| Cirrus-sac W | 113 | 141 | 125 | 87 | 103 | 93 |  | 98 | 186 | 147 | 102 |
| Ovary to posterior end | 392 | 541 | 454 | 233 | 311 | 282 | 368 | 300 | 568 | 420 | 301 |
| Ovary to posterior end as % BL | 30.1 | 35.5 | 33.3 | 31.5 | 34.7 | 33.3 | 35.9 | 31.5 | 40.2 | 34.9 | 32.3 |
| Ovary L | 91 | 130 | 112 | 66 | 94 | 76 | 115 | 44 | 110 | 80 | 55 |
| Ovary W | 78 | 113 | 96 | 57 | 70 | 62 | 82 | 44 | 90 | 72 | 48 |
| Vitelline follicles to anterior end | 184 | 246 | 207 | 136 | 163 | 150 | 154 | 161 | 251 | 201 | 152 |
| Vitelline follicles to anterior end as % BL | 13.4 | 16.2 | 15.3 | 15.9 | 18.6 | 17.8 | 15.0 | 14.0 | 21.3 | 16.9 | 16.3 |
| Vitelline follicles to posterior end | 400 | 478 | 437 | 219 | 327 | 258 | 306 | 280 | 508 | 396 | 282 |
| Vitelline follicles to posterior end as % BL | 30.9 | 33.8 | 32.2 | 25.4 | 36.5 | 30.6 | 29.9 | 29.9 | 35.9 | 33.0 | 30.3 |
| Length vitelline field | 619 | 815 | 713 | 367 | 485 | 436 | 564 | 497 | 737 | 597 | 497 |
| Length vitelline field as % BL | 51.4 | 53.0 | 52.5 | 47.6 | 56.3 | 51.7 | 55.1 | 45.1 | 53.8 | 50.1 | 53.4 |
| Egg L | 68 | 90 | 79 | 70 | 81 | 77 | 79 | 70 | 88 | 77 |  |
| Egg W | 35 | 46 | 41 | 42 | 44 | 43 | 43 | 39 | 49 | 45 |  |
| Excretory vesicle to anterior end | 147 | 205 | 167 | 135 | 155 | 147 | 157 | 125 | 196 | 167 | 124 |
| Excretory vesicle to anterior end as % BL | 11.5 | 13.3 | 12.3 | 15.9 | 18.3 | 17.4 | 15.3 | 11.5 | 18.3 | 14.1 | 13.3 |
