# Supplementary material for: A world of taxonomic pain: cryptic species, inexplicable host-specificity, and host-induced morphological variation among species of Bivesicula Yamaguti, 1934 (Trematoda: Bivesiculidae) from Indo-Pacific Holocentridae, Muraenidae and Serranidae
Source: Parasitology. 2022 Mar 10;149(6):831–53. doi: 10.1017/S0031182022000282 (PMC10090613; doi:10.1017/S0031182022000282)
Supplement: Supplementary file 1 [file S0031182022000282sup001.zip › S0031182022000282sup002.docx]

**Supplementary Table 2**. Measurement *Bivesicula sheni* n. sp.

| Host family | Serranidae | | | Serranidae | Holocentridae | Serranidae | | | Serranidae | | |
| --- | --- | --- | --- | --- | --- | --- | --- | --- | --- | --- | --- |
| Host species | *E. fasciatus* | | | *E. undulatostriatus* | *S. rubrum* | *E. cyanopodus* | | | *E. fasciatus* | | |
| Locality | Heron Island | | | Heron Island | Heron Island | Lizard Island | | | Lizard Island | | |
| n | 15 | | | 1 | 1 | 3 | | | 15 | | |
|  | **Min** | **Max** | **Mean** |  |  | **Min** | **Max** | **Mean** | **Min** | **Max** | **Mean** |
| Body L | 1103 | 1640 | 1374 | 1739 | 859 | 1154 | 1171 | 1162 | 955 | 1493 | 1270 |
| Body W | 302 | 687 | 477 | 448 | 416 | 375 | 419 | 390 | 342 | 534 | 444 |
| Body L / Body W | 2.38 | 3.74 | 2.95 | 3.88 | 2.06 | 2.77 | 3.11 | 2.99 | 2.29 | 3.56 | 2.88 |
| Pharynx L | 94 | 173 | 126 | 123 | 103 | 137 | 165 | 152 | 98 | 167 | 135 |
| Pharynx W | 106 | 164 | 135 | 148 | 107 | 133 | 168 | 154 | 119 | 166 | 141 |
| Pharynx L / Pharynx W | 0.76 | 1.06 | 0.93 | 0.83 | 0.96 | 0.92 | 1.03 | 0.99 | 0.78 | 1.04 | 0.95 |
| Oesophagus | 61 | 191 | 150 | 203 | 119 | 117 | 133 | 123 | 78 | 187 | 133 |
| Caeca to posterior end | 227 | 380 | 304 | 453 | 225 | 278 | 309 | 289 | 210 | 453 | 327 |
| Caeca to posterior end as % BL | 17.6 | 25.9 | 22.2 | 26.0 | 26.2 | 23.7 | 26.8 | 24.8 | 21.4 | 32.9 | 25.7 |
| Testis L | 120 | 254 | 176 | 193 | 100 | 143 | 170 | 152 | 132 | 211 | 178 |
| Testis W | 94 | 237 | 160 | 162 | 91 | 112 | 168 | 139 | 122 | 205 | 160 |
| Testis to anterior end | 784 | 1198 | 985 | 1255 | 631 | 783 | 873 | 830 | 701 | 1032 | 878 |
| Testis to anterior end as % BL | 67.9 | 75.5 | 71.8 | 72.2 | 73.5 | 67.9 | 74.6 | 71.4 | 65.0 | 73.4 | 69.2 |
| Cirrus-sac to anterior end | 544 | 847 | 677 | 870 | 446 | 423 | 592 | 531 | 458 | 700 | 584 |
| Cirrus-sac to anterior end as % BL | 46.1 | 52.8 | 49.3 | 50.0 | 51.9 | 36.4 | 50.6 | 45.7 | 41.4 | 51.4 | 46.1 |
| Cirrus-sac L | 170 | 371 | 264 | 277 | 170 | 198 | 279 | 243 | 217 | 319 | 270 |
| Cirrus-sac W | 95 | 188 | 144 | 133 | 93 | 118 | 146 | 135 | 129 | 204 | 161 |
| Ovary to posterior end | 320 | 578 | 414 | 549 | 263 | 319 | 374 | 341 | 251 | 491 | 410 |
| Ovary to posterior end as % BL | 25.5 | 35.3 | 30.0 | 31.6 | 30.6 | 27.5 | 32.4 | 29.3 | 26.3 | 37.1 | 32.2 |
| Ovary L | 75 | 164 | 105 | 103 | 80 | 83 | 93 | 89 | 70 | 123 | 97 |
| Ovary W | 66 | 136 | 94 | 91 | 70 | 73 | 91 | 81 | 64 | 100 | 82 |
| Vitelline follicles to anterior end | 149 | 379 | 247 | 284 | 186 | 210 | 231 | 220 | 154 | 268 | 203 |
| Vitelline follicles to anterior end as % BL | 12.2 | 23.2 | 17.9 | 16.3 | 21.7 | 18.1 | 20.0 | 18.9 | 13.2 | 18.0 | 16.0 |
| Vitelline follicles to posterior end | 315 | 473 | 396 | 534 | 223 | 325 | 360 | 342 | 253 | 488 | 414 |
| Vitelline follicles to posterior end as % BL | 25.4 | 33.5 | 28.8 | 30.7 | 26.0 | 28.2 | 30.7 | 29.4 | 26.5 | 36.4 | 32.5 |
| Length vitelline field | 558 | 890 | 731 | 921 | 450 | 592 | 610 | 600 | 529 | 793 | 653 |
| Length vitelline field as % BL | 50.6 | 61.6 | 53.3 | 53.0 | 52.4 | 50.6 | 52.5 | 51.6 | 46.0 | 57.1 | 51.5 |
| Egg L | 74 | 87 | 80 | 76 | 80 | 73 | 78 | 75 | 69 | 83 | 74 |
| Egg W | 36 | 50 | 44 | 51 | 33 | 41 | 48 | 45 | 32 | 52 | 42 |
| Excretory vesicle to anterior end | 115 | 223 | 167 | 154 | 137 | 151 | 187 | 167 | 139 | 203 | 164 |
| Excretory vesicle to anterior end as % BL | 9.9 | 17.2 | 12.2 | 8.9 | 15.9 | 12.9 | 16.1 | 14.4 | 11.3 | 15.4 | 13.0 |

**Supplementary Table 2 (cont.)**. Measurement *Bivesicula sheni* n. sp.

| Host family | Serranidae | | | Serranidae | | | Serranidae | Serranidae | | | Holocentridae | | |
| --- | --- | --- | --- | --- | --- | --- | --- | --- | --- | --- | --- | --- | --- |
| Host species | *E. maculata* | | | *E. merra* | | | *E. ongus* | *E. quoyanus* | | | *S. caudimaculatum* | | |
| Locality | Lizard Island | | | Lizard Island | | | Lizard Island | Lizard I. | | | Lizard I. | | |
| n | 12 | | | 9 | | | 1 | 24 | | | 5 | | |
|  | **Min** | **Max** | **Mean** | **Min** | **Max** | **Mean** |  | **Min** | **Max** | **Mean** | **Min** | **Max** | **Mean** |
| Body L | 816 | 1324 | 1150 | 1039 | 1947 | 1532 | 1721 | 833 | 1672 | 1357 | 864 | 1070 | 959 |
| Body W | 317 | 536 | 451 | 343 | 690 | 584 | 510 | 321 | 607 | 457 | 340 | 488 | 415 |
| Body L / Body W | 2.21 | 3.05 | 2.57 | 1.96 | 3.03 | 2.64 | 3.37 | 2.38 | 3.67 | 2.97 | 2.19 | 2.54 | 2.33 |
| Pharynx L | 108 | 166 | 131 | 94 | 191 | 154 | 126 | 78 | 143 | 118 | 90 | 112 | 100 |
| Pharynx W | 110 | 174 | 140 | 103 | 233 | 153 | 153 | 84 | 188 | 138 | 108 | 125 | 116 |
| Pharynx L / Pharynx W | 0.71 | 1.41 | 0.95 | 0.59 | 1.41 | 1.03 | 0.82 | 0.63 | 1.12 | 0.86 | 0.80 | 0.94 | 0.87 |
| Oesophagus | 78 | 175 | 126 | 120 | 268 | 184 | 104 | 74 | 204 | 139 | 129 | 168 | 144 |
| Caeca to posterior end | 196 | 336 | 281 | 287 | 618 | 467 | 455 | 239 | 528 | 387 | 194 | 266 | 229 |
| Caeca to posterior end as % BL | 20.8 | 28.4 | 24.4 | 25.0 | 33.5 | 30.2 | 26.4 | 20.6 | 33.8 | 28.4 | 22.3 | 25.8 | 23.8 |
| Testis L | 91 | 197 | 137 | 121 | 323 | 228 | 205 | 97 | 298 | 190 | 104 | 172 | 139 |
| Testis W | 74 | 152 | 122 | 94 | 269 | 196 | 198 | 84 | 279 | 173 | 94 | 163 | 133 |
| Testis to anterior end | 591 | 902 | 811 | 715 | 1331 | 999 | 1195 | 594 | 1126 | 923 | 635 | 762 | 693 |
| Testis to anterior end as % BL | 63.6 | 75.3 | 70.6 | 61.6 | 69.5 | 65.3 | 69.4 | 62.0 | 76.3 | 68.4 | 71.0 | 73.5 | 72.3 |
| Cirrus-sac to anterior end | 429 | 663 | 546 | 515 | 939 | 708 | 851 | 411 | 806 | 644 | 426 | 566 | 491 |
| Cirrus-sac to anterior end as % BL | 42.2 | 55.0 | 47.7 | 39.4 | 52.0 | 46.2 | 49.4 | 42.1 | 54.8 | 47.7 | 48.9 | 53.8 | 51.2 |
| Cirrus-sac L | 151 | 322 | 235 | 159 | 327 | 275 | 272 | 158 | 333 | 253 | 180 | 206 | 191 |
| Cirrus-sac W | 69 | 149 | 125 | 81 | 183 | 153 | 114 | 84 | 198 | 147 | 99 | 122 | 111 |
| Ovary to posterior end | 241 | 411 | 347 | 363 | 698 | 564 | 552 | 261 | 613 | 457 | 255 | 332 | 288 |
| Ovary to posterior end as % BL | 26.5 | 33.3 | 30.1 | 32.2 | 39.7 | 36.9 | 32.1 | 25.7 | 39.3 | 33.4 | 29.1 | 31.1 | 30.0 |
| Ovary L | 39 | 89 | 72 | 68 | 164 | 132 | 147 | 62 | 134 | 100 | 66 | 93 | 83 |
| Ovary W | 29 | 77 | 63 | 59 | 136 | 107 | 128 | 45 | 130 | 84 | 61 | 77 | 73 |
| Vitelline follicles to anterior end | 150 | 241 | 197 | 167 | 316 | 249 | 210 | 129 | 263 | 208 | 148 | 217 | 186 |
| Vitelline follicles to anterior end as % BL | 12.9 | 21.0 | 17.2 | 14.7 | 18.6 | 16.3 | 12.2 | 12.8 | 20.7 | 15.6 | 17.1 | 21.3 | 19.4 |
| Vitelline follicles to posterior end | 216 | 412 | 343 | 341 | 694 | 531 | 530 | 288 | 624 | 455 | 220 | 313 | 268 |
| Vitelline follicles to posterior end as % BL | 20.3 | 34.6 | 29.8 | 30.6 | 36.3 | 34.5 | 30.8 | 27.5 | 39.6 | 33.4 | 24.1 | 29.9 | 27.9 |
| Length vitelline field | 412 | 752 | 610 | 531 | 945 | 752 | 981 | 416 | 973 | 694 | 458 | 540 | 504 |
| Length vitelline field as % BL | 47.7 | 59.1 | 53.0 | 45.4 | 52.8 | 49.2 | 57.0 | 45.2 | 58.2 | 51.0 | 50.5 | 57.5 | 52.7 |
| Egg L | 70 | 79 | 75 | 73 | 86 | 78 | 75 | 68 | 84 | 78 | 72 | 78 | 75 |
| Egg W | 33 | 52 | 43 | 37 | 50 | 44 | 46 | 37 | 51 | 45 | 39 | 47 | 43 |
| Excretory vesicle to anterior end | 126 | 189 | 162 | 127 | 303 | 208 | 180 | 120 | 211 | 177 | 148 | 165 | 159 |
| Excretory vesicle to anterior end as % BL | 10.5 | 16.7 | 14.1 | 11.1 | 17.8 | 13.5 | 10.5 | 10.4 | 16.9 | 13.3 | 15.1 | 18.9 | 16.7 |

**Supplementary Table 2 (cont.)**. Measurement *Bivesicula sheni* n. sp.

| Host family | Holocentridae | | |
| --- | --- | --- | --- |
| Host species | *S. spiniferum* | | |
| Locality | Lizard Island | | |
| n | 10 | | |
|  | **Min** | **Max** | **Mean** |
| Body L | 684 | 1125 | 941 |
| Body W | 351 | 571 | 454 |
| Body L / Body W | 1.82 | 2.50 | 2.08 |
| Pharynx L | 57 | 139 | 91 |
| Pharynx W | 81 | 134 | 106 |
| Pharynx L / Pharynx W | 0.70 | 1.04 | 0.85 |
| Oesophagus | 99 | 143 | 129 |
| Caeca to posterior end | 183 | 348 | 249 |
| Caeca to posterior end as % BL | 20.3 | 31.2 | 26.3 |
| Testis L | 82 | 207 | 132 |
| Testis W | 78 | 190 | 110 |
| Testis to anterior end | 476 | 762 | 649 |
| Testis to anterior end as % BL | 64.5 | 71.6 | 69.2 |
| Cirrus-sac to anterior end | 334 | 504 | 426 |
| Cirrus-sac to anterior end as % BL | 40.5 | 48.8 | 45.5 |
| Cirrus-sac L | 120 | 267 | 199 |
| Cirrus-sac W | 79 | 180 | 123 |
| Ovary to posterior end | 207 | 422 | 316 |
| Ovary to posterior end as % BL | 30.3 | 37.8 | 33.4 |
| Ovary L | 52 | 145 | 85 |
| Ovary W | 47 | 103 | 70 |
| Vitelline follicles to anterior end | 125 | 234 | 170 |
| Vitelline follicles to anterior end as % BL | 14.7 | 22.7 | 18.2 |
| Vitelline follicles to posterior end | 215 | 374 | 291 |
| Vitelline follicles to posterior end as % BL | 28.4 | 35.8 | 30.9 |
| Length vitelline field | 330 | 635 | 480 |
| Length vitelline field as % BL | 41.5 | 56.4 | 50.9 |
| Egg L | 64 | 80 | 73 |
| Egg W | 33 | 49 | 42 |
| Excretory vesicle to anterior end | 113 | 180 | 151 |
| Excretory vesicle to anterior end as % BL | 14.1 | 20.6 | 16.1 |
