# Supplementary material for: A world of taxonomic pain: cryptic species, inexplicable host-specificity, and host-induced morphological variation among species of Bivesicula Yamaguti, 1934 (Trematoda: Bivesiculidae) from Indo-Pacific Holocentridae, Muraenidae and Serranidae
Source: Parasitology. 2022 Mar 10;149(6):831–53. doi: 10.1017/S0031182022000282 (PMC10090613; doi:10.1017/S0031182022000282)
Supplement: Supplementary file 1 [file S0031182022000282sup001.zip › S0031182022000282sup003.docx]

**Supplementary Table 3**. Measurements *Bivesicula polynesiensis* n. sp.

| Host family | Serranidae | | | Holocentridae | Holocentridae | | | Holocentridae | | | Serranidae | | |
| --- | --- | --- | --- | --- | --- | --- | --- | --- | --- | --- | --- | --- | --- |
| Host locality | *E. fasciatus* | | | *N. sammara* | *S. diadema* | | | *S. spiniferum* | | | *C. urodeta* | | |
| Locality | FP: Australs | | | FP: Gambiers | FP: Gambiers | | | FP: Gambiers | | | FP: Moorea | | |
| n | 10 | | | 1 | 9 | | | 6 | | | 3 | | |
|  | **Min** | **Max** | **Mean** |  | **Min** | **Max** | **Mean** | **Min** | **Max** | **Mean** | **Min** | **Max** | **Mean** |
| Body L | 954 | 1166 | 1081 | 1091 | 870 | 966 | 925 | 1042 | 1166 | 1094 | 1245 | 1277 | 1264 |
| Body W | 399 | 465 | 434 | 521 | 383 | 446 | 409 | 569 | 658 | 611 | 428 | 579 | 514 |
| Body L / Body W | 2.28 | 2.71 | 2.49 | 2.09 | 2.00 | 2.49 | 2.27 | 1.68 | 2.05 | 1.79 | 2.21 | 2.97 | 2.50 |
| Pharynx L | 141 | 177 | 167 | 137 | 117 | 138 | 127 | 131 | 148 | 140 | 152 | 188 | 171 |
| Pharynx W | 141 | 168 | 158 | 133 | 109 | 129 | 121 | 133 | 165 | 146 | 130 | 158 | 142 |
| Pharynx L / Pharynx W | 0.96 | 1.21 | 1.06 | 1.03 | 1.01 | 1.10 | 1.05 | 0.88 | 1.03 | 0.97 | 1.09 | 1.36 | 1.21 |
| Oesophagus | 43 | 71 | 58 | 82 | 69 | 114 | 90 | 116 | 156 | 139 | 55 | 67 | 63 |
| Caeca to posterior end | 278 | 372 | 332 | 343 | 224 | 300 | 257 | 307 | 367 | 341 | 296 | 346 | 326 |
| Caeca to posterior end as % BL | 25.8 | 33.5 | 30.8 | 31.4 | 23.5 | 31.5 | 27.7 | 29.2 | 32.9 | 31.2 | 23.2 | 27.8 | 25.8 |
| Testis L | 152 | 185 | 168 | 240 | 159 | 207 | 179 | 148 | 223 | 197 | 175 | 206 | 192 |
| Testis W | 129 | 166 | 154 | 219 | 126 | 179 | 154 | 148 | 208 | 178 | 133 | 205 | 174 |
| Testis to anterior end | 629 | 747 | 702 | 704 | 592 | 677 | 629 | 677 | 757 | 703 | 797 | 942 | 873 |
| Testis to anterior end as % BL | 61.9 | 68.1 | 65.0 | 64.5 | 66.3 | 71.0 | 68.0 | 61.4 | 66.6 | 64.3 | 64.0 | 73.8 | 69.0 |
| Cirrus-sac to anterior end | 382 | 513 | 446 | 416 | 369 | 435 | 406 | 416 | 497 | 458 | 561 | 670 | 612 |
| Cirrus-sac to anterior end as % BL | 38.3 | 45.9 | 41.3 | 38.1 | 39.5 | 46.2 | 43.9 | 39.9 | 43.8 | 41.8 | 45.1 | 52.5 | 48.3 |
| Cirrus-sac L | 220 | 293 | 251 | 258 | 185 | 236 | 212 | 230 | 274 | 248 | 230 | 268 | 248 |
| Cirrus-sac W | 139 | 183 | 150 | 181 | 115 | 170 | 140 | 158 | 272 | 195 | 141 | 146 | 144 |
| Ovary to posterior end | 367 | 459 | 409 | 412 | 272 | 336 | 303 | 378 | 442 | 399 | 366 | 448 | 410 |
| Ovary to posterior end as % BL | 34.4 | 42.1 | 37.8 | 37.8 | 30.9 | 35.9 | 32.8 | 34.6 | 37.9 | 36.5 | 28.7 | 36.0 | 32.5 |
| Ovary L | 70 | 101 | 88 | 116 | 87 | 102 | 94 | 93 | 106 | 101 | 107 | 125 | 113 |
| Ovary W | 55 | 82 | 70 | 98 | 63 | 87 | 78 | 75 | 99 | 86 | 80 | 100 | 92 |
| Vitelline follicles to anterior end | 139 | 197 | 167 | 152 | 127 | 161 | 146 | 138 | 195 | 170 | 182 | 197 | 190 |
| Vitelline follicles to anterior end as % BL | 12.8 | 18.3 | 15.5 | 13.9 | 13.6 | 17.2 | 15.8 | 12.8 | 17.2 | 15.5 | 14.3 | 15.8 | 15.0 |
| Vitelline follicles to posterior end | 369 | 461 | 407.7 | 380 | 298 | 364 | 317 | 375 | 431 | 399.5 | 395 | 472 | 445 |
| Vitelline follicles to posterior end as % BL | 34.4 | 42.3 | 37.7 | 34.8 | 31.3 | 38.2 | 34.3 | 35.6 | 38.1 | 36.5 | 30.9 | 37.7 | 35.2 |
| Length vitelline field | 370 | 574 | 507 | 559 | 431 | 512 | 462 | 486 | 552 | 525 | 579 | 700 | 629 |
| Length vitelline field as % BL | 38.8 | 52.0 | 46.8 | 51.2 | 47.2 | 53.0 | 49.9 | 45.9 | 51.3 | 48.0 | 46.5 | 54.8 | 49.7 |
| Egg L | 64 | 83 | 73 | 78 | 78 | 83 | 81 | 73 | 82 | 77 | 78 | 83 | 81 |
| Egg W | 40 | 48 | 42 | 50 | 38 | 51 | 46 | 46 | 48 | 47 | 39 | 43 | 41 |
| Excretory vesicle to anterior end | 162 | 185 | 172 | 172 | 135 | 173 | 160 | 160 | 203 | 185 | 150 | 191 | 167 |
| Excretory vesicle to anterior end as % BL | 14.2 | 17.2 | 15.9 | 15.8 | 14.4 | 19.4 | 17.4 | 14.1 | 19.3 | 16.9 | 11.8 | 15.3 | 13.2 |

**Supplementary Table 3 (cont.)**. Measurements *Bivesicula polynesiensis* n. sp.

| Host family | Holocentridae | | |
| --- | --- | --- | --- |
| Host species | *S. microstoma* | | |
| Locality | FP: Moorea | | |
| n | 1 | | |
|  | **Min** | **Max** | **Mean** |
| Body L | 977 | 1006 | 991 |
| Body W | 540 | 609 | 567 |
| Body L / Body W | 1.60 | 1.86 | 1.75 |
| Pharynx L | 98 | 120 | 108 |
| Pharynx W | 114 | 133 | 126 |
| Pharynx L / Pharynx W | 0.82 | 0.90 | 0.86 |
| Oesophagus | 67 | 97 | 81 |
| Caeca to posterior end | 286 | 331 | 303 |
| Caeca to posterior end as % BL | 28.9 | 32.9 | 30.5 |
| Testis L | 160 | 228 | 204 |
| Testis W | 153 | 209 | 186 |
| Testis to anterior end | 626 | 645 | 634 |
| Testis to anterior end as % BL | 62.2 | 65.1 | 63.9 |
| Cirrus-sac to anterior end | 370 | 380 | 374 |
| Cirrus-sac to anterior end as % BL | 37.0 | 38.3 | 37.7 |
| Cirrus-sac L | 228 | 264 | 249 |
| Cirrus-sac W | 148 | 172 | 163 |
| Ovary to posterior end | 350 | 402 | 371 |
| Ovary to posterior end as % BL | 35.3 | 40.0 | 37.4 |
| Ovary L | 105 | 111 | 107 |
| Ovary W | 83 | 96 | 88 |
| Vitelline follicles to anterior end | 127 | 137 | 131 |
| Vitelline follicles to anterior end as % BL | 12.8 | 13.6 | 13.2 |
| Vitelline follicles to posterior end | 320 | 364 | 337 |
| Vitelline follicles to posterior end as % BL | 32.8 | 36.2 | 34.0 |
| Length vitelline field | 505 | 537 | 523 |
| Length vitelline field as % BL | 50.2 | 54.2 | 52.8 |
| Egg L | 78 | 80 | 79 |
| Egg W | 43 | 52 | 47 |
| Excretory vesicle to anterior end | 158 | 168 | 163 |
| Excretory vesicle to anterior end as % BL | 15.9 | 16.8 | 16.5 |
