# Supplementary material for: A world of taxonomic pain: cryptic species, inexplicable host-specificity, and host-induced morphological variation among species of Bivesicula Yamaguti, 1934 (Trematoda: Bivesiculidae) from Indo-Pacific Holocentridae, Muraenidae and Serranidae
Source: Parasitology. 2022 Mar 10;149(6):831–53. doi: 10.1017/S0031182022000282 (PMC10090613; doi:10.1017/S0031182022000282)
Supplement: Supplementary file 1 [file S0031182022000282sup001.zip › S0031182022000282sup004.docx]

**Supplementary Table 4**. *Bivesicula* *cephalopholicola* n. sp. measurements

| Host family | Serranidae | | | Serranidae | | | Serranidae | | |
| --- | --- | --- | --- | --- | --- | --- | --- | --- | --- |
| Host species | *C. boenak* | | | *C. microprion* | | | *C. boenak* | | |
| Locality | Lizard Island | | | Lizard Island | | | New Caledonia | | |
| n | 3 | | | 6 | | | 3 | | |
|  | **Min** | **Max** | **Mean** | **Min** | **Max** | **Mean** | **Min** | **Max** | **Mean** |
| Body L | 999 | 1429 | 1201 | 884 | 1113 | 969 | 1133 | 1562 | 1304 |
| Body W | 311 | 366 | 343 | 284 | 380 | 322 | 402 | 550 | 456 |
| Body L / Body W | 3.21 | 3.90 | 3.48 | 2.65 | 3.32 | 3.03 | 2.59 | 3.20 | 2.87 |
| Pharynx L | 103 | 165 | 128 | 98 | 122 | 110 | 110 | 143 | 121 |
| Pharynx W | 109 | 161 | 133 | 112 | 139 | 126 | 121 | 165 | 138 |
| Pharynx L / Pharynx W | 0.88 | 1.02 | 0.95 | 0.72 | 0.98 | 0.88 | 0.85 | 0.93 | 0.88 |
| Oesophagus | 92 | 130 | 116 | 41 | 101 | 73 | 117 | 146 | 133 |
| Caeca to posterior end | 256 | 397 | 305 | 231 | 294 | 262 | 336 | 527 | 382 |
| Caeca to posterior end as % BL | 22.4 | 27.8 | 25.3 | 26.1 | 28.2 | 27.1 | 25.9 | 33.7 | 29.2 |
| Testis L | 127 | 154 | 143 | 86 | 105 | 96 | 134 | 187 | 163 |
| Testis W | 105 | 153 | 125 | 81 | 98 | 89 | 104 | 183 | 144 |
| Testis to anterior end | 771 | 899 | 837 | 623 | 836 | 691 | 774 | 1042 | 889 |
| Testis to anterior end as % BL | 62.9 | 77.2 | 70.6 | 66.3 | 75.1 | 71.2 | 65.8 | 69.8 | 68.2 |
| Cirrus-sac to anterior end | 536 | 721 | 629 | 437 | 599 | 502 | 522 | 750 | 635 |
| Cirrus-sac to anterior end as % BL | 50.5 | 53.7 | 52.6 | 48.2 | 53.8 | 51.7 | 46.1 | 50.0 | 48.6 |
| Cirrus-sac L | 136 | 221 | 171 | 135 | 152 | 142 | 199 | 280 | 223 |
| Cirrus-sac W | 81 | 108 | 91 | 75 | 90 | 81.5 | 104 | 158 | 123 |
| Ovary to posterior end | 274 | 417 | 344 | 275 | 338 | 302 | 370 | 624 | 457 |
| Ovary to posterior end as % BL | 27.4 | 29.2 | 28.6 | 28.7 | 33.9 | 31.3 | 31.8 | 39.9 | 34.8 |
| Ovary L | 89 | 110 | 97 | 65 | 86 | 73 | 74 | 166 | 102 |
| Ovary W | 65 | 102 | 82 | 52 | 74 | 66 | 65 | 121 | 86 |
| Vitelline follicles to anterior end | 184 | 253 | 214 | 158 | 204 | 190 | 171 | 228 | 195 |
| Vitelline follicles to anterior end as % BL | 17.5 | 18.4 | 17.9 | 17.4 | 21.9 | 19.7 | 13.2 | 16.1 | 15.0 |
| Vitelline follicles to posterior end | 274 | 408 | 327 | 263 | 309 | 282 | 371 | 471 | 415 |
| Vitelline follicles to posterior end as % BL | 25.6 | 28.6 | 27.2 | 26.8 | 31.3 | 29.2 | 29.0 | 35.7 | 32.0 |
| Length vitelline field | 541 | 768 | 659 | 425 | 600 | 497 | 562 | 885 | 694 |
| Length vitelline field as % BL | 53.7 | 57.0 | 55.0 | 48.1 | 54.1 | 51.2 | 48.2 | 56.7 | 52.9 |
| Egg L | 75 | 78 | 76.3 | 74.0 | 81.0 | 79 | 80 | 92 | 84 |
| Egg W | 45 | 50 | 48 | 37 | 49 | 44 | 36 | 47 | 43 |
| Excretory vesicle to anterior end | 122 | 195 | 155 | 122 | 149 | 137 | 133 | 175 | 155 |
| Excretory vesicle to anterior end as % BL | 12.2 | 13.6 | 12.8 | 12.4 | 15.1 | 14.1 | 10.4 | 13.5 | 12.0 |
