# Supplementary material for: A world of taxonomic pain: cryptic species, inexplicable host-specificity, and host-induced morphological variation among species of Bivesicula Yamaguti, 1934 (Trematoda: Bivesiculidae) from Indo-Pacific Holocentridae, Muraenidae and Serranidae
Source: Parasitology. 2022 Mar 10;149(6):831–53. doi: 10.1017/S0031182022000282 (PMC10090613; doi:10.1017/S0031182022000282)
Supplement: Supplementary file 1 [file S0031182022000282sup001.zip › S0031182022000282sup005.docx]

**Supplementary Table 5**. *Bivesicula palauensis* Shimazu & Machida, 1995 measurements.

| Host family | Serranidae | | Serranidae |
| --- | --- | --- | --- |
| Host species | *V. albimarginatus* | | *E. morrhua* |
| Locality | Okinawa | | New Caledonia |
| n | 2 | | 1 |
| Body L | 1717 | 1872 | 2390 |
| Body W | 694 | 749 | 1253 |
| Body L / Body W | 2.47 | 2.50 | 1.91 |
| Pharynx L | 76 | 109 | 137 |
| Pharynx W | 104 | 109 | 180 |
| Pharynx L / Pharynx W | 0.73 | 1.00 | 0.76 |
| Oesophagus | 124 | 165 | 275 |
| Caeca to posterior end | 562 | 602 | 372 |
| Caeca to posterior end as % BL | 32.2 | 32.7 | 15.6 |
| Testis L | 284 | 323 | 490 |
| Testis W | 218 | 254 | 472 |
| Testis to anterior end | 1043 | 1053 | 1273 |
| Testis to anterior end as % BL | 55.7 | 61.3 | 53.3 |
| Cirrus-sac to anterior end | 646 | 657 | 808 |
| Cirrus-sac to anterior end as % BL | 35.1 | 37.6 | 33.8 |
| Cirrus-sac L | 260 | 282 |  |
| Cirrus-sac W | 166 | 183 |  |
| Ovary to posterior end | 663 | 822 | 1102 |
| Ovary to posterior end as % BL | 38.6 | 43.9 | 46.1 |
| Ovary L | 170 | 175 | 251 |
| Ovary W | 168 | 172 | 236 |
| Vitelline follicles to anterior end | 194 | 221 | 337 |
| Vitelline follicles to anterior end as % BL | 11.3 | 11.8 | 14.1 |
| Vitelline follicles to posterior end | 212 | 218 | 230 |
| Vitelline follicles to posterior end as % BL | 11.3 | 12.7 | 9.6 |
| Length vitelline field | 1305 | 1439 | 1823 |
| Length vitelline field as % BL | 76.0 | 76.9 | 76.3 |
| Egg L | 86 | 93 | 90 |
| Egg W | 44 | 48 | 48 |
| Excretory vesicle to anterior end | 145 | 215 | 238 |
| Excretory vesicle to anterior end as % BL | 8.4 | 11.5 | 10.0 |
