# Supplementary material for: A world of taxonomic pain: cryptic species, inexplicable host-specificity, and host-induced morphological variation among species of Bivesicula Yamaguti, 1934 (Trematoda: Bivesiculidae) from Indo-Pacific Holocentridae, Muraenidae and Serranidae
Source: Parasitology. 2022 Mar 10;149(6):831–53. doi: 10.1017/S0031182022000282 (PMC10090613; doi:10.1017/S0031182022000282)
Supplement: Supplementary file 1 [file S0031182022000282sup001.zip › S0031182022000282sup006.docx]

**Supplementary Table 6**. *Bivesicula gymnothoracis* Shimazu & Machida, 1995 measurements.

| Host family | Serranidae | | | Muraenidae | | |
| --- | --- | --- | --- | --- | --- | --- |
| Host species | *E. fasciatus* | | | *G. kidako* | | |
| Locality | Minabe, Japan | | | Minabe, Japan | | |
| n | 15 | | | 9 | | |
|  | **Min** | **Max** | **Mean** | **Min** | **Max** | **Mean** |
| Body L | 1041 | 1356 | 1212 | 1292 | 2988 | 2496 |
| Body W | 520 | 749 | 668 | 659 | 1551 | 1247 |
| Body L / Body W | 2 | 2 | 2 | 2 | 3 | 2 |
| Pharynx L | 73 | 92 | 84 | 78 | 176 | 139 |
| Pharynx W | 85 | 119 | 100 | 108 | 220 | 173 |
| Pharynx L / Pharynx W | 1 | 1 | 1 | 1 | 1 | 1 |
| Oesophagus | 69 | 156 | 99 | 138 | 287 | 214 |
| Caeca to posterior end | 351 | 567 | 458 | 391 | 1092 | 923 |
| Caeca to posterior end as % BL | 31.0 | 42.1 | 37.7 | 30.3 | 43.5 | 36.6 |
| Testis L | 199 | 267 | 225 | 288 | 688 | 533 |
| Testis W | 169 | 255 | 207 | 258 | 539 | 466 |
| Testis to anterior end | 582 | 771 | 687 | 747 | 1688 | 1378 |
| Testis to anterior end as % BL | 53.2 | 61.8 | 56.8 | 53.4 | 57.8 | 55.3 |
| Cirrus-sac to anterior end | 336 | 486 | 417 | 495 | 971 | 767 |
| Cirrus-sac to anterior end as % BL | 29.8 | 38.5 | 34.5 | 27.4 | 38.3 | 31.1 |
| Cirrus-sac L | 246 | 315 | 272 | 280 | 701 | 615 |
| Cirrus-sac W | 157 | 204 | 180 | 172 | 491 | 407 |
| Ovary to posterior end | 394 | 585 | 511 | 482 | 1277 | 1089 |
| Ovary to posterior end as % BL | 34.8 | 44.6 | 42.1 | 37.3 | 48.4 | 43.3 |
| Ovary L | 101 | 136 | 121 | 126 | 323 | 231 |
| Ovary W | 76 | 127 | 107 | 108 | 246 | 195 |
| Vitelline follicles to anterior end | 90 | 178 | 141 | 123 | 312 | 213 |
| Vitelline follicles to anterior end as % BL | 7.4 | 14.8 | 11.7 | 6.6 | 11.3 | 8.5 |
| Vitelline follicles to posterior end | 239 | 471 | 335 | 249 | 896 | 675 |
| Vitelline follicles to posterior end as % BL | 20.5 | 35.4 | 27.7 | 19.3 | 31.2 | 26.5 |
| Length vitelline field | 595 | 902 | 736 | 920 | 1918 | 1609 |
| Length vitelline field as % BL | 54.3 | 68.2 | 60.6 | 57.8 | 72.8 | 64.9 |
| Egg L | 76 | 101 | 85 | 83 | 107 | 95 |
| Egg W | 45 | 55 | 49 | 44 | 58 | 50 |
| Excretory vesicle to anterior end | 165 | 259 | 205 | 194 | 408 | 352 |
| Excretory vesicle to anterior end as % BL | 14.6 | 19.5 | 16.9 | 12.4 | 15.5 | 14.2 |
