# Supplementary material for: A world of taxonomic pain: cryptic species, inexplicable host-specificity, and host-induced morphological variation among species of Bivesicula Yamaguti, 1934 (Trematoda: Bivesiculidae) from Indo-Pacific Holocentridae, Muraenidae and Serranidae
Source: Parasitology. 2022 Mar 10;149(6):831–53. doi: 10.1017/S0031182022000282 (PMC10090613; doi:10.1017/S0031182022000282)
Supplement: Supplementary file 1 [file S0031182022000282sup001.zip › S0031182022000282sup007.docx]

**Supplementary Table 7**. *Bivesicula novaecaledoniensis* n. sp*.* measurements.

| Host family | Serranidae | | | Serranidae | | |
| --- | --- | --- | --- | --- | --- | --- |
| Host species | *E. chlorostigma* | | | *E. fasciatus* | | |
| Locality | New Caledonia | | | New Caledonia | | |
| n | 19 | | | 3 | | |
|  | **Min** | **Max** | **Mean** | **Min** | **Max** | **Mean** |
| Body L | 1242 | 2046 | 1622 | 997 | 1372 | 1176 |
| Body W | 566 | 1002 | 782 | 521 | 704 | 610 |
| Body L / Body W | 2 | 2 | 2 | 2 | 2 | 2 |
| Pharynx L | 67 | 116 | 91 | 77 | 94 | 85 |
| Pharynx W | 87 | 129 | 112 | 93 | 116 | 103 |
| Pharynx L / Pharynx W | 1 | 1 | 1 | 1 | 1 | 1 |
| Oesophagus | 116 | 252 | 202 | 126 | 179 | 153 |
| Caeca to posterior end | 311 | 844 | 519 | 328 | 420 | 384 |
| Caeca to posterior end as % BL | 0.0 | 41.4 | 30.2 | 30.6 | 34.9 | 32.8 |
| Testis L | 201 | 374 | 281 | 166 | 294 | 219 |
| Testis W | 154 | 343 | 255 | 150 | 282 | 196 |
| Testis to anterior end | 748 | 1167 | 965 | 602 | 780 | 687 |
| Testis to anterior end as % BL | 55.5 | 64.6 | 59.7 | 56.9 | 60.4 | 58.6 |
| Cirrus-sac to anterior end | 450 | 735 | 590 | 380 | 453 | 408 |
| Cirrus-sac to anterior end as % BL | 30.9 | 42.6 | 36.4 | 28.6 | 39.1 | 35.3 |
| Cirrus-sac L | 210 | 414 | 332 | 217 | 347 | 275 |
| Cirrus-sac W | 151 | 292 | 227 | 141 | 243 | 176 |
| Ovary to posterior end | 504 | 921 | 684 | 395 | 567 | 471 |
| Ovary to posterior end as % BL | 37.1 | 45.1 | 42.0 | 38.8 | 41.3 | 39.9 |
| Ovary L | 86 | 188 | 139 | 110 | 142 | 124 |
| Ovary W | 74 | 170 | 115 | 84 | 130 | 107 |
| Vitelline follicles to anterior end | 135 | 294 | 202 | 106 | 193 | 147 |
| Vitelline follicles to anterior end as % BL | 10.0 | 15.2 | 12.5 | 10.6 | 14.1 | 12.3 |
| Vitelline follicles to posterior end | 297 | 603 | 417 | 305 | 351 | 328 |
| Vitelline follicles to posterior end as % BL | 19.2 | 30.1 | 25.5 | 22.2 | 32.8 | 28.4 |
| Length vitelline field | 810 | 1241 | 1003 | 564 | 874 | 701 |
| Length vitelline field as % BL | 57.8 | 68.8 | 61.9 | 56.6 | 63.7 | 59.2 |
| Egg L | 74 | 95 | 86 | 85 | 91 | 88 |
| Egg W | 38 | 50 | 45 | 44 | 47 | 46 |
| Excretory vesicle to anterior end | 154 | 333 | 251 | 151 | 238 | 189 |
| Excretory vesicle to anterior end as % BL | 0.0 | 18.9 | 14.6 | 15.1 | 17.3 | 16.0 |
