# Supplementary figures and images for: A world of taxonomic pain: cryptic species, inexplicable host-specificity, and host-induced morphological variation among species of Bivesicula Yamaguti, 1934 (Trematoda: Bivesiculidae) from Indo-Pacific Holocentridae, Muraenidae and Serranidae
Source: Parasitology. 2022 Mar 10;149(6):831–53. doi: 10.1017/S0031182022000282 (PMC10090613; doi:10.1017/S0031182022000282)

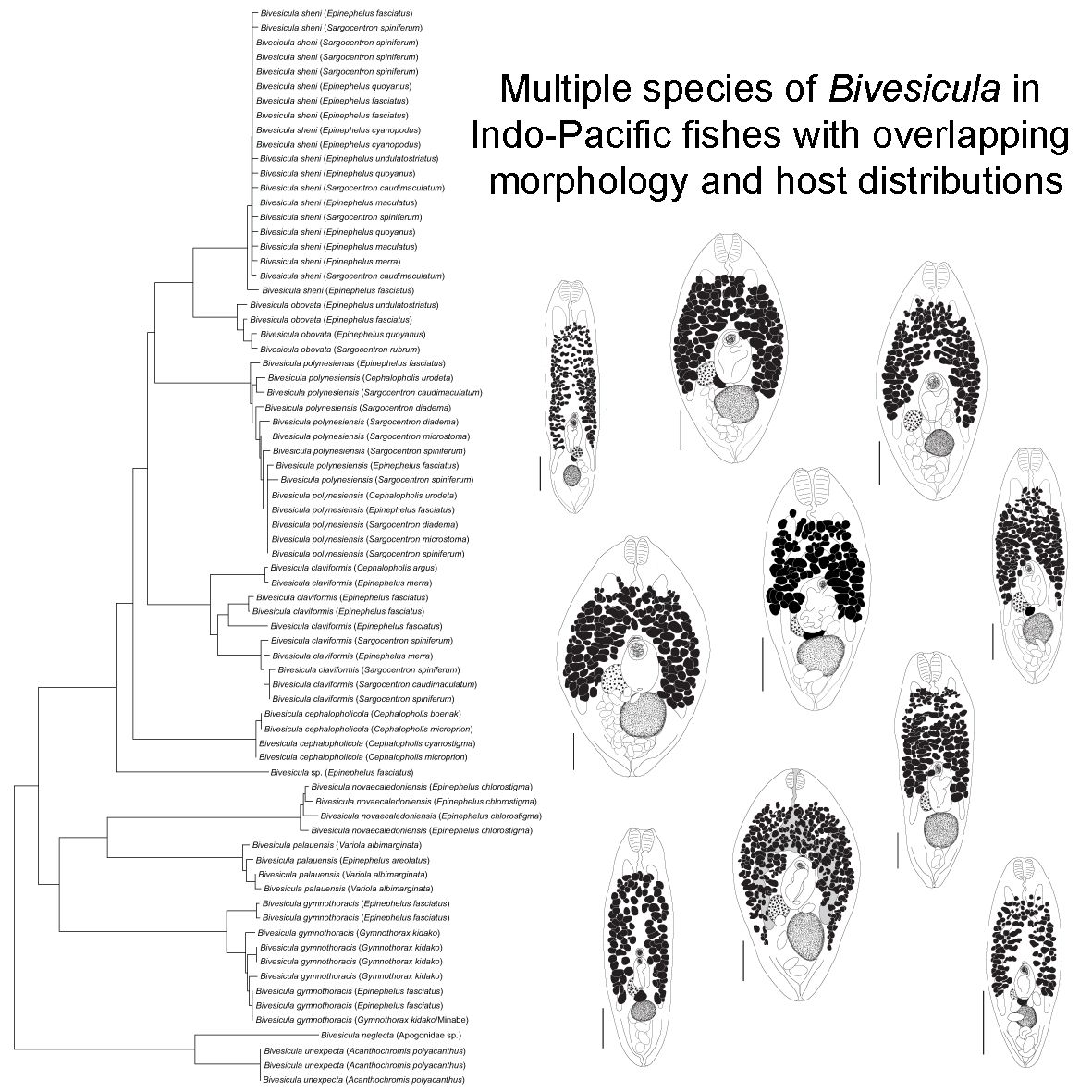

Supplement: Supplementary file 1 [file S0031182022000282sup001.zip › S0031182022000282sup008.tif]
